# The Ancestral KEAP1-NRF Pathway in Amphioxus *Branchiostoma japonicum*: Implications for the Evolution of Antioxidant Defense System

**DOI:** 10.3390/ijms26073427

**Published:** 2025-04-06

**Authors:** Weichen Li, Xiaoqian Liang, Keyu Xiang, Hongyan Li, Yu Zhang

**Affiliations:** 1 College of Marine Life Sciences, Institute of Evolution & Marine Biodiversity, Ocean University of China, Qingdao 266003, China; lwc15106557901@outlook.com (W.L.);; 2 Key Laboratory of Evolution & Marine Biodiversity (Ministry of Education), Ocean University of China, Qingdao 266003, China; 3 Laboratory for Marine Biology and Biotechnology, Qingdao Marine Science and Technology Center, Qingdao 266237, China

**Keywords:** amphioxus, oxidative stress, NRF1, NRF2, KEAP1

## Abstract

The Kelch-like ECH-associated protein 1 (KEAP1)/Nuclear factor E2-related factor 2 (NRF2) pathway is a key mechanism that responds to oxidative stress and xenobiotic stimuli in vertebrates. However, knowledge of its evolutionary origins remains limited. In this study, we identify the ancestral homologues of KEAP1 and NRF (BjKEAP1 and BjNRF) in cephalochordate amphioxus (*Branchiostoma japonicum*). BjNRF uniquely combines the feature domains of vertebrates NRF1 and NRF2, marking it as an evolutionary intermediate. High expression levels of *Bjkeap*1 and *Bjnrf* in the gill, hepatic cecum, and intestine highlight their roles in environmental defense at key interface tissues. Functional studies reveal that BjKEAP1 regulates the cytoplasmic localization of BjNRF. Typical NRF2 activator sulforaphane (SFN) induces its nuclear translocation and significantly elevates the transcriptional expression of BjNRF and phase II detoxification enzymes. Moreover, exposure to the environmental toxin Benzo[a]pyrene (BaP) activates this stress response system. These findings bridge critical gaps in our understanding of this pathway in basal chordates and offer new insights into the evolutionary trajectory of the KEAP1-NRF system. Furthermore, this study highlights crucial implications for the conservation of amphioxus in deteriorating marine environments.

## 1. Introduction

Nuclear factor E2-related factor 2 (NRF2), a member of the Cap ‘n’ collar-basic leucine zipper (CNC-bZIP) family, plays an essential role in maintaining redox balance, metabolism, and normal development and regulating cellular stress responses [1,2,3,4]. The activity of NRF2 is negatively regulated by the Kelch-like ECH-associated protein 1 (KEAP1) through the E3 ubiquitin-proteasome system. In vertebrates, the KEAP1-NRF2 signaling pathway serves as a crucial anti-stress mechanism, activating the expression of various genes in response to intracellular oxidative stress and xenobiotic stimuli [5,6,7,8]. Under homeostatic conditions, KEAP1 binds NRF2 to the ubiquitin ligase complex, promoting its ubiquitination and subsequent degradation [9,10]. During oxidative stress, KEAP1, acting as a primary stress sensor, undergoes covalent modifications on reactive cysteine residues in its intervening region (IVR). These modifications alter KEAP1’s protein conformation, inhibiting its interaction with NRF2 and thereby reducing the degradation of NRF2 [10]. Accumulated NRF2 translocates to the nucleus, where it heterodimerizes with small Maf (sMaf) proteins and binds to the antioxidant response element (ARE) of target genes [11,12,13].

NRF2 homologues are found in both invertebrates and vertebrates, playing a crucial role in the regulation of cellular homeostasis. However, the characteristics of NRF2 homologues differ significantly between these groups [14,15,16,17,18,19,20,21,22,23]. In invertebrates, the mechanisms underlying NRF2’s involvement in antioxidant defense are notably distinct. For instance, in nematodes, the absence of KEAP1 leads to the activation of the NRF homologue SKN1 primarily through phosphorylation by kinases such as AKT, PMK-1/p38, and GSK-3 [24,25,26]. Additionally, SKN1 binds to DNA as a monomer, a feature that sets it apart from vertebrate NRF2, which forms a heterodimer with sMaf [2]. In Drosophila, the NRF homologue, Cnc, is negatively regulated by KEAP1, similar to vertebrate NRF2. However, Cnc also exhibits unique functions by generating multiple isoforms, thereby expanding its functional versatility [2,27,28]. These findings suggest that while NRF homologues serve as an antioxidant system across metazoan organisms, the regulatory mechanisms display notable variations among different species.

As an evolutionary transition species between invertebrates and vertebrates, the cephalochordate amphioxus is recognized as an ideal organism for studying vertebrate evolution [29,30,31,32]. Despite its significance, the KEAP1-NRF2 pathway remains poorly characterized in basal chordates, presenting a valuable opportunity to investigate the evolutionary origins of this pathway. In this study, we identified the opening reading frame (ORF) sequences of amphioxus orthologues of vertebrate *nrf*1/2 and *keap*1, naming them *Bjnrf* and *Bjkeap*1, respectively. Phylogenetic and detailed domain structure analyses suggest that BjNRF and BjKEAP1 represent ancestral forms, and BjNRF incorporates feature domain motifs of both NRF1 and NRF2. Moreover, *Bjnrf* and *Bjkeap*1 showed high expression levels in the gill, hepatic cecum, and intestine tissues constantly exposed to environmental stimuli. Furthermore, our findings demonstrate that the BjKEAP1-BjNRF interaction exists in the basal chordate amphioxus, playing a critical role in antioxidant defense. This research provides evolutionary insights into the KEAP1-NRF2 pathway and contributes to the understanding of antioxidant defense mechanisms in basal chordates, aiding amphioxus conservation in increasingly polluted marine environments.

## 2. Results

### 2.1. Characterization and Phylogenetic Analysis of B. japonicum NRF

**Figure 1:**
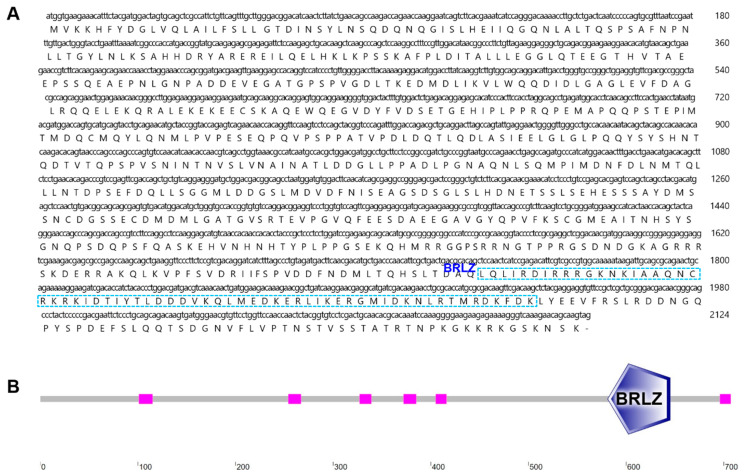
The sequence and secondary structure of *B. japonicum* NRF (BjNRF). (**A**) The nucleotide and deduced amino acid sequences of BjNRF. The BRLZ (basic region leucine zipper) domain was labeled and indicated in blue boxes. (**B**) The prediction of secondary structure of BjNRF using SMART (https://smart.embl.de/, accessed on 23 September 2023).

In this study, we identified an ORF of the *B. japonicum nrf* gene, consisting of 2124 base pairs, which encodes a protein with 707 amino acids (Figure 1A). The deduced protein contains a basic region leucine zipper (BRLZ) domain at the C-terminal region, indicating it belongs to the bZIP superfamily (Figure 1B). The tertiary structure of the *B. japonicum* NRF protein closely resembled that of human and zebrafish NRF2 (Supplementary Figure S1). These findings confirm that we successfully obtained the target sequence, which we designated as *Bjnrf*.

**Figure 2:**
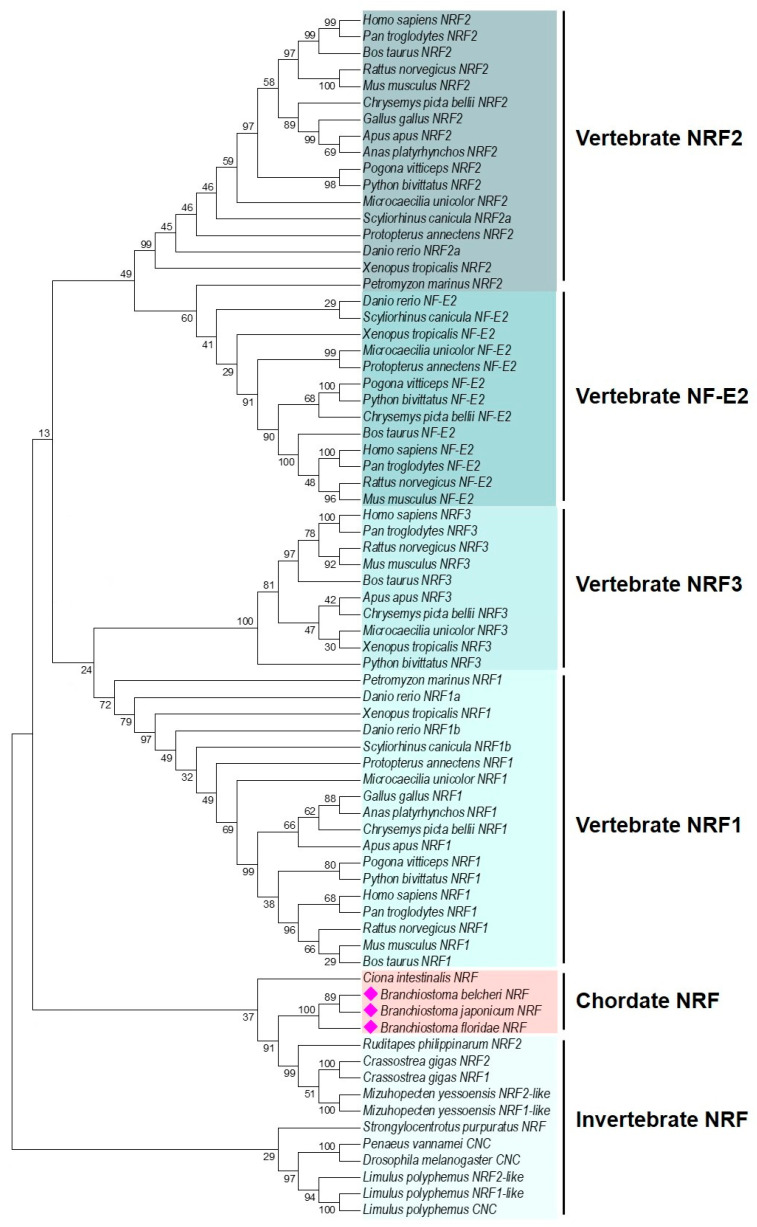
Phylogenetic tree of NRF homologues. The phylogenetic tree was constructed by MEGA 7.0 using the amino acid-based Maximum Likelihood (ML) algorithm. The reliability of each node was estimated by bootstrapping with 1000 replications. Amphioxus NRF are indicated in red diamonds. The accession numbers for sequences used are listed in Table S2 of Supplementary Materials.

Vertebrates typically possess multiple NRF paralogues (NRF1, NRF2, NRF3, and NF-E2); a search of the amphioxus genome in the NCBI database revealed the presence of only a single NRF gene locus. Phylogenetic analysis showed that the four vertebrate NRF paralogues clustered into distinct groups, whereas NRF homologues from three amphioxus species grouped together with invertebrate NRFs at the basal position of the tree (Figure 2 and Supplementary Figure S2), suggesting amphioxus *nrf* represents the ancestral form.

### 2.2. Domain Structure Analysis of BjNRF

**Figure 3:**
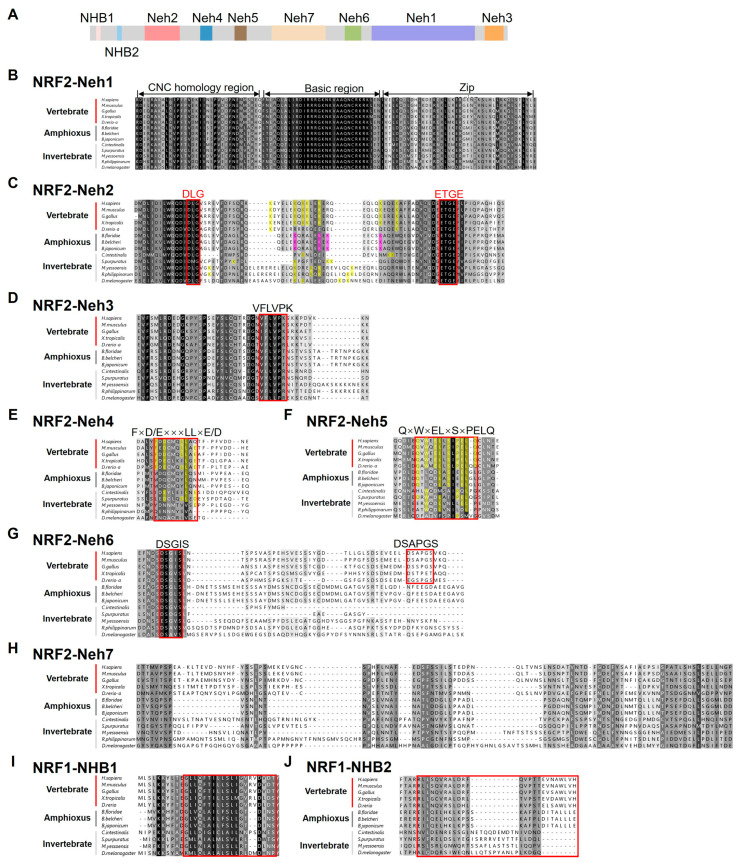
Domain structure analysis of the BjNRF and NRF homologues of various species. (**A**) BjNRF is composed of Neh1-7 domains and NHB1/2 domains. (**B**–**H**) Sequence alignment of NRF2 Neh1-7 domains among human (*H. sapiens*), mouse (*M. musculus*), chicken (*G. gallus*), frog (*X. tropicalis*), zebrafish (*D. rerio*), amphioxus (*B. floridae*, *B. belcheri*, and *B. japonicum*), ascidian (*C. intestinalis*), sea urchin (*S. purpuratus*), scallop (*M. yessoensis*), clam (*R. philippinarum*), and fruit fly (*D. melanogaster*). (**I**,**J**) Sequence alignment of NRF1 NHB1/2 domains among amphioxus and model species. Critical motifs are indicated in red boxes. The lysine residues between DLG and ETGE motif of BjNRF are shaded in pink and those of other species are shaded in yellow. Basic amino acid residues in the FxD/ExxxLLxE/D motif and the QxWxELxSxPELQ motif are shown in the yellow background. Accession numbers for sequences used are listed in Table S2 of Supplementary Materials.

To preliminarily explore the function of amphioxus NRF, we conducted an in-depth analysis of the domain structure of BjNRF. Vertebrate NRF1/2 are divided into seven distinct regions, known as NRF2–ECH homology (Neh) domains 1–7, based on sequence conservation and functional characteristics [33]. As shown in Figure 3A, the Neh1-7 domains were also identified within BjNRF, along with the N-terminal homology box (NHB) domain, which serves as an endoplasmic reticulum binding site.

The Neh1 domain, also termed the CNC-bZip (Cap’n’ Collar-basic leucine zipper) domain, consists of three functional regions: the CNC homology region, the basic region, and the leucine zipper region [33]. Sequence alignment showed high conservation of amino acids across these three regions in both BjNRF and the NRF proteins of other species examined (Figure 3B).

The Neh2 domain, essential for NRF2’s binding to KEAP1, contains two critical motifs, DLG and ETGE. In general, seven lysine (K) residues typically lie between these motifs, acting as key sites for ubiquitination [34]. Sequence alignment revealed that BjNRF contains the essential DLG and ETGE motifs, along with key lysine residues (Figure 3C). Notably, BjNRF has four lysine residues between these motifs, a number that differs from the seven lysine residues in vertebrate NRF2 and the two found in vertebrate NRF1 (Supplementary Figure S3). However, considering the relative positions of these lysine residues, BjNRF showed more similarity to vertebrate NRF2.

The Neh3, Neh4, Neh5, and Neh7 domains are recognized as transcriptional regulatory regions [35]. In the Neh3 domain, the VFLVPK motif, which is essential for full transcriptional activity [2], was conserved in BjNRF as well as in the NRF proteins of other analyzed species (Figure 3D). The Neh4 domain contains the TRAM (FxD/ExxxL) motif, considered a transcriptional adaptor [36]. However, vertebrate-homologous TRAM motifs were absent in the invertebrates examined, including amphioxus (Figure 3E). In the Neh5 domain, the QxWxELxxSxPELQ motif—a highly conserved region in vertebrate NRF2 orthologues [37]—was found to be less conserved in BjNRF (Figure 3F). Despite this, the sequence of BjNRF Neh4 and Neh5 exhibited greater similarity to those in NRF2 than to those in NRF1 (Supplementary Figure S4). Lastly, the Neh7 domain, known to repress NRF2 transcriptional activity by interacting with retinoid X receptor α (RXRα) [35], appeared less conserved across species examined (Figure 3H).

The Neh6 domain of vertebrate NRF2 plays a role in the negative regulation of NRF2 through a KEAP1-independent mechanism, containing two conserved motifs, DSGIS and DSAPGS [38]. As shown in Figure 3G, the DSGIS motif was present in invertebrate NRFs, including BjNRF, while the DSAPGS motif was absent.

In addition to the Neh1-7 domains, an NHB domain was also identified at the N-terminus of BjNRF, which is responsible for binding to the endoplasmic reticulum and is unique to vertebrate NRF1/3 [2,39]. Sequence alignment showed that NHB1, rather than NHB2, was conserved between BjNRF and vertebrate NRF1 (Figure 3I).

### 2.3. Characterization and Phylogenetic Analysis of B. japonicum KEAP1

**Figure 4:**
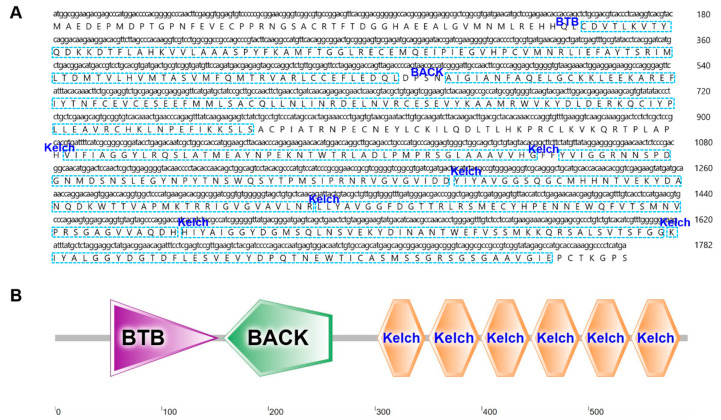
The sequence and structure of *B. japonicum* KEAP1 (BjKEAP1). (**A**) The nucleotide and deduced amino acid sequences of BjKEAP1. The conserved BTB (Broad-Complex, Tramtrack, and Bric-a-brac), BACK (BTB and C-terminal Kelch) domains, and six Kelch motifs are labeled and indicated in blue boxes. (**B**) The prediction of secondary structure of BjKEAP1 using SMART (https://smart.embl.de/, accessed on 23 September 2023).

**Figure 5:**
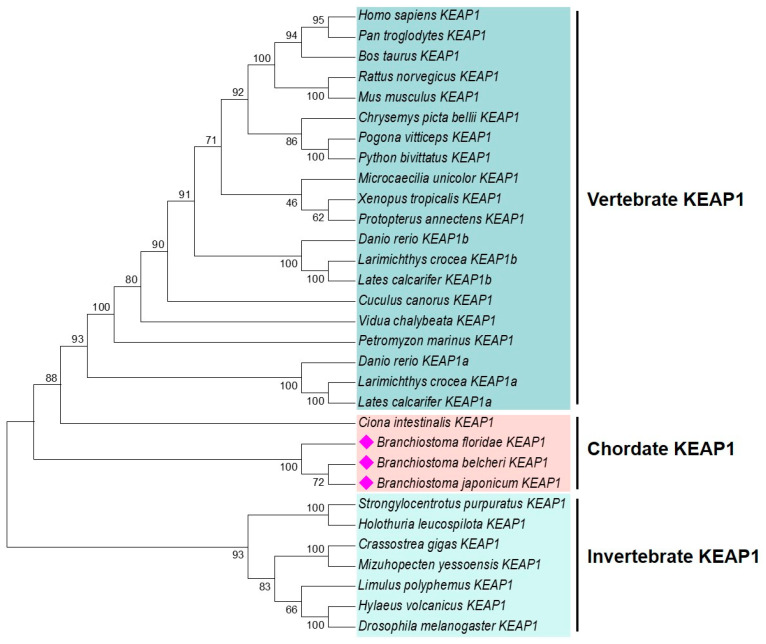
Phylogenetic tree of KEAP1 homologues. The phylogenetic tree was constructed by MEGA 7.0 using the amino acid-based Maximum Likelihood (ML) algorithm. The reliability of each node was estimated by bootstrapping with 1000 replications. Amphioxus KEAP1 are indicated in red diamonds. The accession numbers for the sequences used are listed in Table S3 of Supplementary Materials.

We obtained the ORF of *B. japonicum keap*1, which is 1782 bp in length and encodes a protein of 593 amino acids (Figure 4A). The deduced protein contains a BTB (Broad-Complex, Tramtrack, and Bric-a-brac) domain, a BACK (BTB and C-terminal Kelch) domain, and six Kelch motifs (Figure 4B). Additionally, the predicted tertiary structure of *B. japonicum* KEAP1 resembled that of humans and zebrafish (Supplementary Figure S5). These findings confirmed that we obtained the correct sequence, which we named *Bjkeap*1. The phylogenetic tree revealed that each taxonomic group formed a distinct cluster, with the amphioxus KEAP1 group positioned between vertebrate and invertebrate branches, well reflecting the phylogeny of the investigated organisms (Figure 5 and Supplementary Figure S6).

### 2.4. Domain Structure Analysis of BjKEAP1

**Figure 6:**
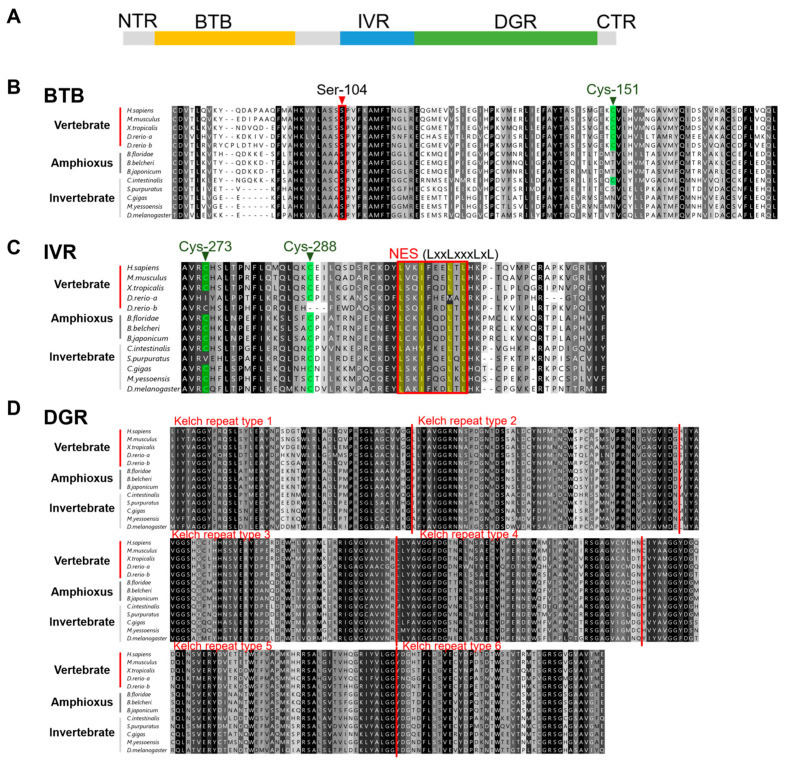
Domain structure analysis of BjKEAP1 and KEAP1 homologues of various species. (**A**) BjKEAP1 is divided into five domains according to its function and conservation, namely NTR (N-terminal region), BTB (broad complex, the tram-track and bric-a-brac), IVR (intervening region), DGR (dihydroxyacetone repeat), and CTR (C-terminal region). (**B**) Amino acid sequence alignment of the BTB domain. The conserved Ser-104 site is shown in a red box. The cysteine residue Cys-151, an amino acid conserved in vertebrates, is shaded in green. (**C**) Amino acid sequence alignment of IVR domain. The conserved cysteine residues Cys-273 and Cys-288 are shown with a green background. The nuclear output signal (LxxLxxxLxL; L is the hydrophobic residue and x is the other amino acid) is indicated in a red box, and within it, hydrophobic residues are shown in yellow. (**D**) Amino acid sequence alignment of the DGR domain. The six conserved Kelch motifs are shown in all species investigated and marked by red lines. The accession numbers for the sequences used are listed in Table S3 of Supplementary Materials.

According to sequence conservation and function, vertebrate KEAP1 is divided into the five regions: the N-terminal region (NTR), the broad complex, the tram-track and bric-a-brac (BTB domain), the intervening region (IVR), the DGR (dihydroxyacetone repeat)/Kelch repeat domain, and the C-terminal region (CTR). Among these, the BTB, IVR, and DGR/Kelch domains are involved in inhibiting NRF2 activity [40]. Detailed structural analysis revealed that BjKEAP1 also contained these five domains, similar to vertebrate KEAP1 (Figure 6A).

The BTB domain plays an important role in KEAP1 homodimer formation, which is necessary for NRF2 ubiquitination [41]. Within this domain, Ser-104 is a crucial site for homodimer formation [42]. Sequence analysis showed that the BTB domain of BjKEAP1 contained the Ser-104 active site, and the amino acid surrounding this site was highly conserved across NRF proteins in the species investigated (Figure 6B). In mammals, Cys-151 within the BTB domain of KEAP1 functions as a sensor site [43,44,45]. Notably, Cys-151 was absent in invertebrate KEAP1, including that of amphioxus, with the exception of ascidians (Figure 6B).

The IVR domain primarily interacts with Cul3 and Rbx1 proteins to form the E3 ligase complex for ubiquitination [46]. Within this domain, Cys-273 and Cys-288 are among the oldest cysteine residues functioning as stress sensors, and the nuclear export signal (NES) is responsible for the cytoplasmic localization of the protein [47]. As shown in Figure 6C, BjKEAP1 contained the IVR domain, as well as Cys-273, Cys-288, and the NES region.

The C-terminal DGR/Kelch domain of KEAP1, a critical region to bind with NRF2, consists of six Kelch repeat motifs [46]. Sequence alignment revealed that the DGR/Kelch domain was highly conserved among the species investigated, including amphioxus (Figure 6D).

### 2.5. Tissue-Specific Expression of Bjnrf and Bjkeap1

**Figure 7:**
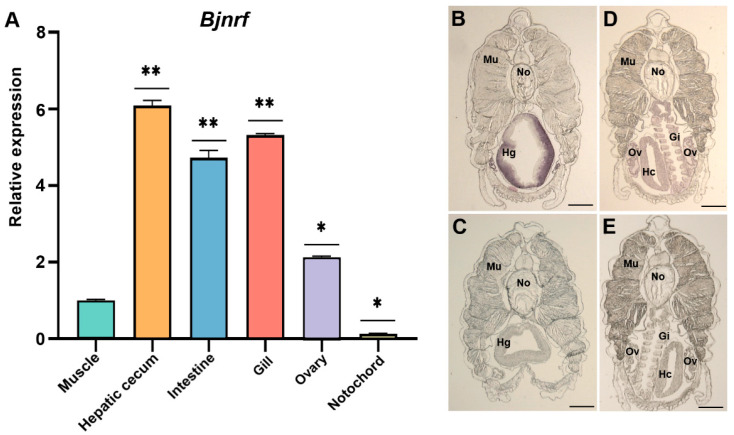
Expression profile of *Bjnrf* in the various tissues. (**A**) The relative expressions of *Bjnrf* were determined in the various tissues, including the muscle, gill, hepatic cecum, ovary, intestine, and notochord, by qRT-PCR. The *β-actin* was chosen as the internal control for normalization. The expression level in the muscle was set at 1. The results shown are mean values ± S.D. (*n* = 3). Asterisks indicate a statistical difference compared to the muscle group. The symbol * indicates *p* < 0.05, ** indicates *p* < 0.01. (**B**,**D**) The relative expression of *Bjnrf* in the different tissues of *B. japonicum* detected by in situ hybridization using *Bjnrf* antisense RNA probes. (**C**,**E**) Control. In situ hybridization was performed using *Bjnrf* sense RNA probes. Gi: gill; Hc: hepatic cecum; Hg: hindgut; Mu: muscle; No: notochord. Ov: ovary. Scale bar: 150 μm.

**Figure 8:**
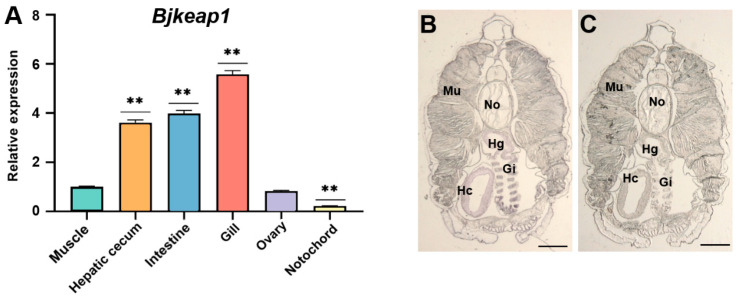
Expression profile of *Bjkeap*1 in the various tissues. (**A**) The relative expression of *Bjkeap*1 gene was determined in the various tissues, including the muscle, gill, hepatic cecum, ovary, intestine, and notochord, by qRT-PCR. The *β-actin* gene was chosen as the internal control for normalization. The expression level in the muscle was set at 1. The results shown are mean values ± S.D. (*n* = 3). Asterisks indicate a statistical difference compared to the muscle group. The symbol ** indicates *p* < 0.01. (**B**) Relative expression of the *Bjkeap*1 gene in the different tissues of *B. japonicum* detected by in situ hybridization using *Bjkeap*1 antisense RNA probes. (**C**) Control. In situ hybridization was performed using *Bjkeap*1 sense RNA probes. Gi: gill; Hc: hepatic cecum; Hg: hindgut; Mu: muscle; No: notochord. Scale bar: 150 μm.

The expression profiles of *Bjnrf* and *Bjkeap*1 were assayed using qRT-PCR and in situ hybridization histochemistry. As shown in Figure 7A and Figure 8A, *Bjnrf* and *Bjkeap*1 mRNA were expressed across all of the tissues examined. However, the transcripts of both genes were notably higher in the hepatic cecum, intestine, and gill, while lower levels were observed in the muscle and notochord. The qRT-PCR findings were consistent with in situ hybridization histochemistry, which revealed significant positive signals for *Bjnrf* and *Bjkeap*1 in the hepatic cecum, intestine, and gill (Figure 7B–E and Figure 8B,C).

### 2.6. Subcellular Localization of BjNRF and BjKEAP1

**Figure 9:**
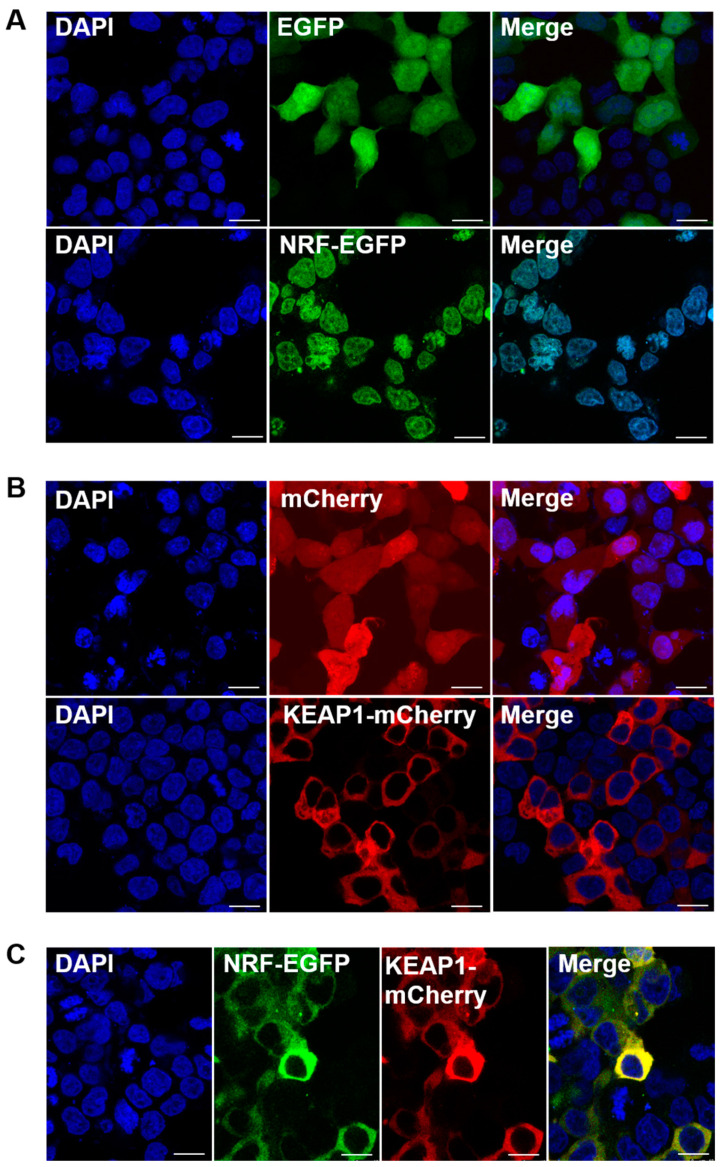
Subcellular localization of *B. japonicum* NRF and KEAP1. (**A**) The HEK293 cells were transiently transfected with pcDNA3.1/BjNRF/EGFP or pcDNA3.1/EGFP. (**B**) The HEK293 cells were transiently transfected with pcDNA3.1/BjKEAP1/mCherry or pcDNA3.1/mCherry. (**C**) The HEK293 cells were transiently transfected with pcDNA3.1/BjNRF/EGFP and pcDNA3.1/BjKEAP1/mCherry. The cells were imaged by fluorescence microscopy. The nucleus was stained by DAPI. Scale bar: 10 μm.

**Figure 10:**
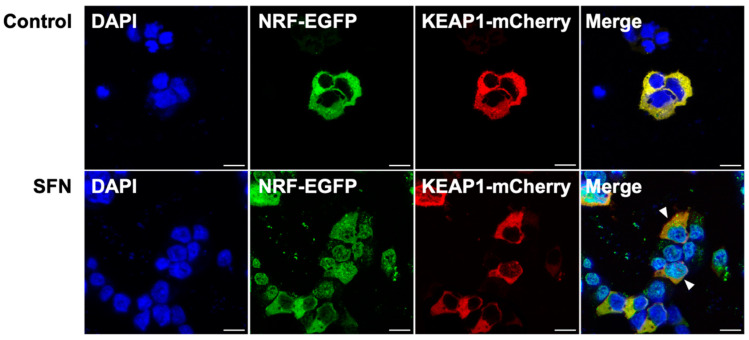
Nuclear translocation of BjNRF upon exposure to SFN. The HEK293 cells were transiently co-transfected with pcDNA3.1/BjNRF/EGFP and pcDNA3.1/BjKEAP1/mCherry were exposed to 20 μM SFN for 6 h. The cells were imaged by fluorescence microscopy. The nucleus was stained by DAPI. White arrowheads indicate cells in which BjNRF translocated into the nucleus. Scale bar: 10 μm.

A subcellular localization assay was conducted using recombinant plasmids (pcDNA3.1-Bjnrf-EGFP and pcDNA3.1-Bjkeap1-mCherry) transfected into the HEK-293 cell line. As shown in Figure 9A, green fluorescence was detected in the nucleus following transfection with pcDNA3.1-Bjnrf-EGFP alone, indicating nuclear localization of BjNRF in the absence of BjKEAP1. Transfection with pcDNA3.1-Bjkeap1-mCherry alone resulted in red fluorescence in the cytoplasm (Figure 9B), indicating the cytoplasmic localization of BjKEAP1. Upon co-transfection with both plasmids, yellow fluorescence was observed in the cytoplasm (Figure 9C), suggesting that BjNRF was retained in the cytoplasm when BjKEAP1 was present. Furthermore, treatment with the NRF2 representative activator sulforaphane (SFN) led to the green fluorescence appearing in the nucleus following co-transfection with both plasmids, indicating the nuclear translocation of BjNRF (Figure 10).

### 2.7. Expression of Bjnrf, Bjkeap1, and Phase II Detoxification Genes in Response to SFN Treatment

To access whether SFN activates the transcriptional levels of *Bjnrf* and its downstream phase II detoxification genes, qRT-PCR was conducted to measure the expression levels of *Bjnrf*, *Bjkeap*1, *glutamatecysteine ligase modifier subunit* (*gclm*), *glutamate-cysteine ligase catalytic* (*gclc*), *glutathione S-transferase P* (*gstp*), and *peroxiredoxin* (*prdx*) in the gills, hepatic cecum, and intestines of *B. japonicum* following treatment with 50 μM SFN for 6, 12, 24, 48, and 96 hours (h).

**Figure 11:**
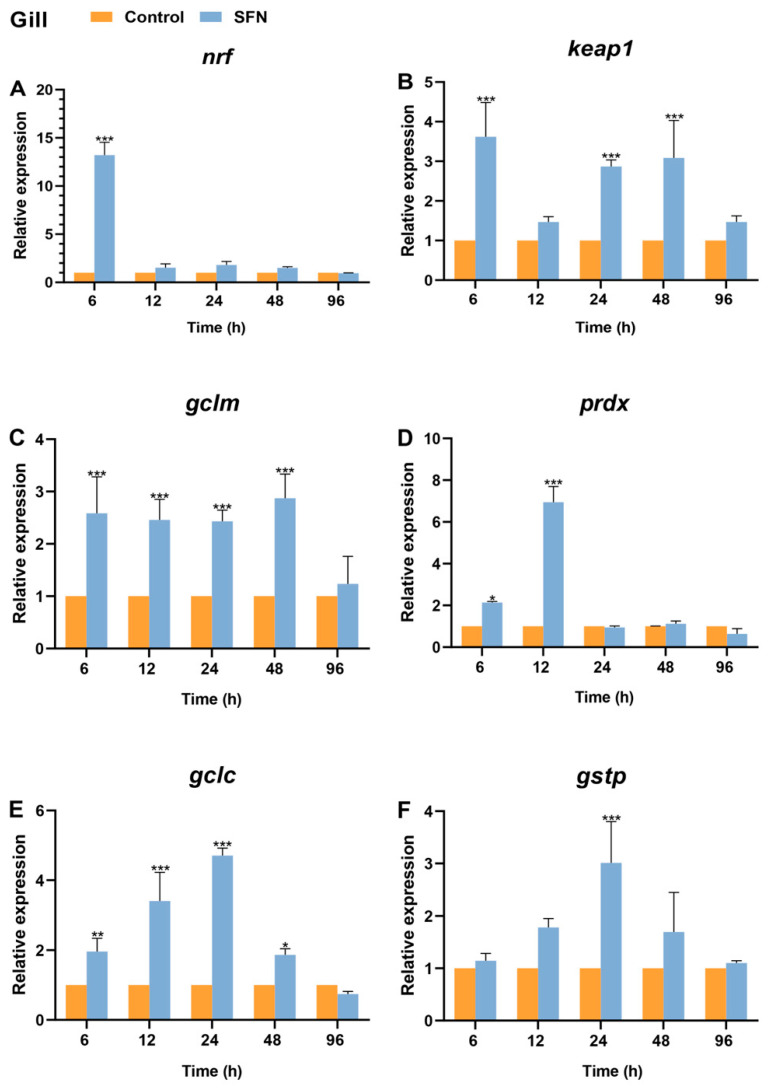
Expression profiles of *Bjnrf*, *Bjkeap*1, and phase II detoxification genes in *B. japonicum* gill after sulforaphane (SFN) treatment. The expression of *Bjnrf* (**A**), *Bjkeap*1 (**B**), *glutamatecysteine ligase modifier subunit* (*gclm*) (**C**), *peroxiredoxin* (*prdx*) (**D**), *glutamate-cysteine ligase catalytic* (*gclc*) (**E**), and *glutathione S-transferase P* (*gstp*) (**F**) was detected by qRT-PCR at 6 h, 12 h, 24 h, 48 h, and 72 h after 50 μM SFN exposure. The *β-actin* gene was used as the internal control for normalization. The symbol * indicates *p* < 0.05, ** indicates *p* < 0.01, and *** indicates *p* < 0.001.

In the gills, *Bjnrf* expression exhibited a dramatic increase at 6 h, returned to basal levels after 12 h, and remained stable until the end of the 96 h treatment (Figure 11A). Although the mRNA level of *Bjkeap*1 also showed a significant increase at 6 h, its fold change was lower than that of *Bjnrf* and displayed a fluctuating pattern from 12 to 96 h (Figure 11B). The expression levels of phase II detoxification genes exhibited a pattern of upregulation followed by subsequent downregulation (Figure 11C,D). Notably, significant upregulation was observed, starting at 6 h for *gclm*, *gclc*, and *prdx*, and at 24 h for *gstp*.

**Figure 12:**
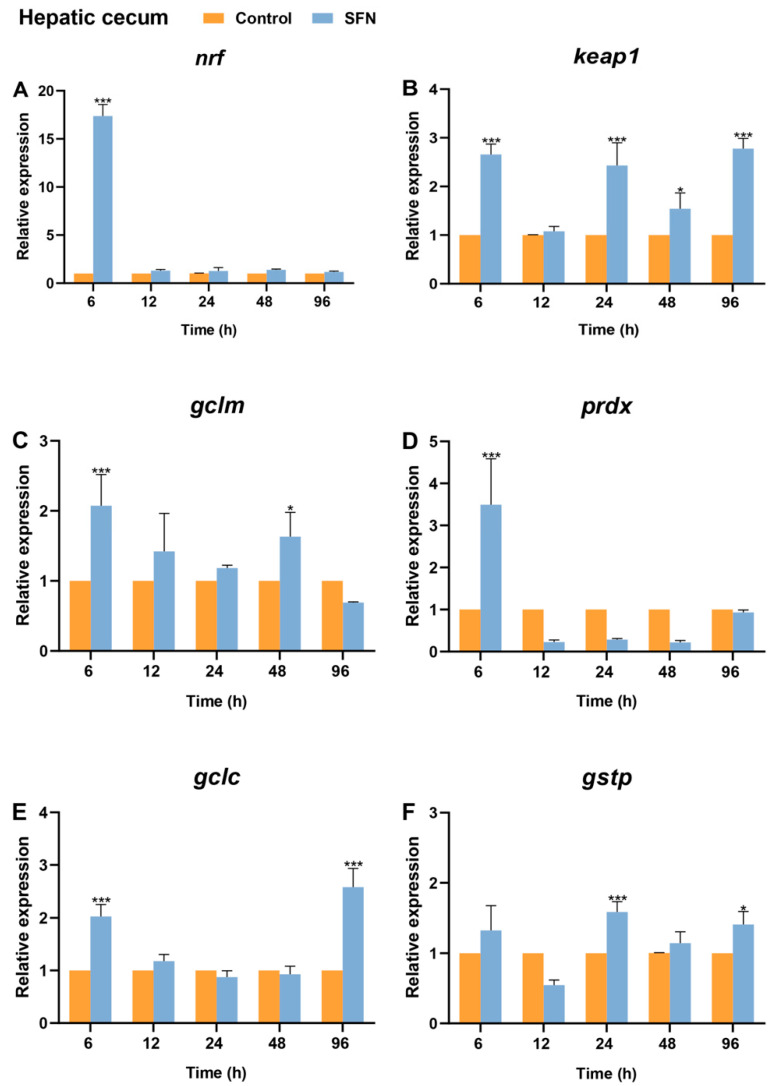
Expression profiles of *Bjnrf*, *Bjkeap*1, and phase II detoxification genes in *B. japonicum* hepatic cecum after sulforaphane (SFN) treatment. The expression of *Bjnrf* (**A**), *Bjkeap*1 (**B**), *glutamatecysteine ligase modifier subunit* (*gclm*) (**C**), *peroxiredoxin* (*prdx*) (**D**), *glutamate-cysteine ligase catalytic* (*gclc*) (**E**), and *glutathione S-transferase P* (*gstp*) (**F**) was detected by qRT-PCR at 6 h, 12 h, 24 h, 48 h, and 72 h after 50 μM SFN exposure. The *β-actin* gene was used as the internal control for normalization. The symbol * indicates *p* < 0.05 and *** indicates *p* < 0.001.

In the hepatic cecum, the expression patterns of *Bjnrf* and *Bjkeap*1 were similar to those observed in the gills (Figure 12A,B). SFN also triggered a strong upregulation of *Bjnrf*, which returned to basal levels by 12 h. Additionally, phase II detoxification genes, including *gclm*, *gclc*, *prdx*, and *gstp*, were also induced, exhibiting significant upregulation at time points similar to those in the gills (Figure 12C–F).

**Figure 13:**
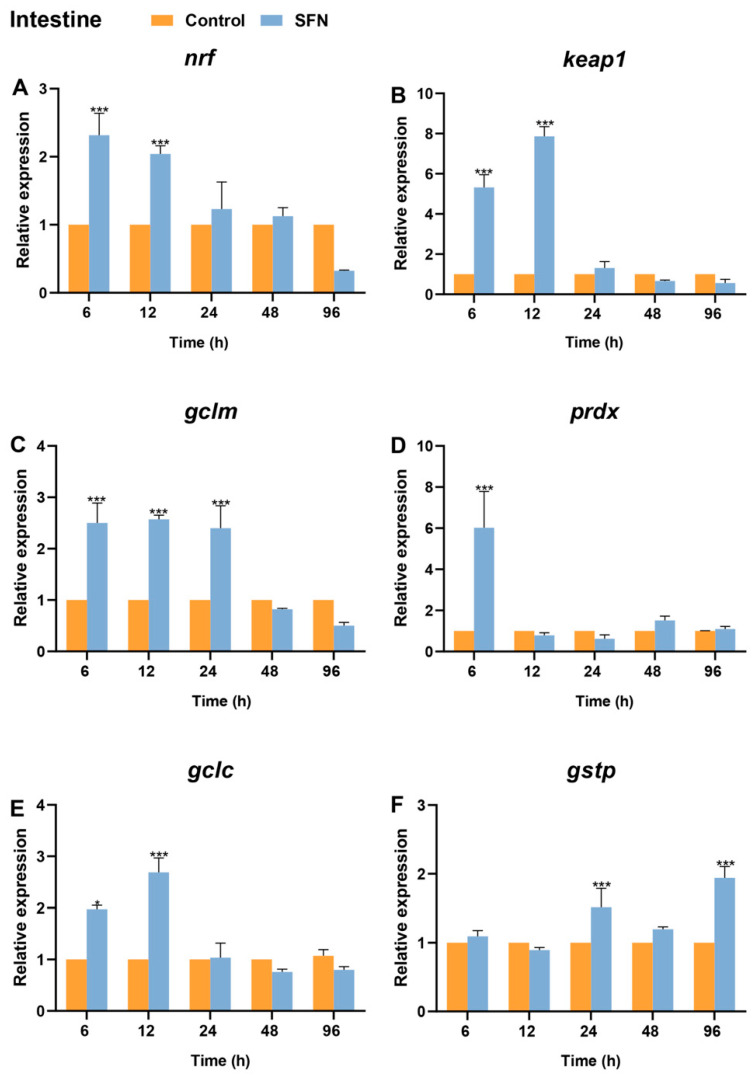
Expression profiles of *Bjnrf*, *Bjkeap*1, and phase II detoxification genes in *B. japonicum* intestine after sulforaphane (SFN) treatment. The expression of *Bjnrf* (**A**), *Bjkeap*1 (**B**), *glutamatecysteine ligase modifier subunit* (*gclm*) (**C**), *peroxiredoxin* (*prdx*) (**D**), *glutamate-cysteine ligase catalytic* (*gclc*) (**E**), and *glutathione S-transferase P* (*gstp*) (**F**) was detected by qRT-PCR at 6 h, 12 h, 24 h, 48 h, and 72 h after 50 μM SFN exposure. The *β-actin* gene was used as the internal control for normalization. The symbol * indicates *p* < 0.05 and *** indicates *p* < 0.001.

Unlike the patterns observed in the gills and hepatic cecum, the significant increase in *Bjnrf* expression in the intestine persisted until 12 h before returning to basal levels (Figure 13A). Additionally, the fold change of *Bjnrf* in the intestine was lower than that in the gills and hepatic cecum. The expression pattern of *Bjkeap*1 was similar to that of *Bjnrf* in the intestine, with a significant increase at 6 and 12 h (Figure 13B). Notably, the fold change of *Bjkeap*1 was higher than that of *Bjnrf*, which contrasted with the patterns observed in the gills and hepatic cecum. Furthermore, phase II detoxification genes in the intestine were also upregulated, following a similar temporal activation pattern to that in the hepatic cecum (Figure 13C–F).

### 2.8. The Expression of Bjnrf, Bjkeap1, and Antioxidant Genes After BaP Exposure

To investigate whether *Bjnrf* and *Bjkeap*1 respond to common ocean pollutants, *B. japonicum* were exposed to Benzo[a]pyrene (BaP) at concentrations of 0, 50, 100, and 200 μg/L. Samples were collected at 24, 48, and 96 h. qRT-PCR was performed to assess the mRNA levels of *Bjnrf*, *Bjkeap*1, and key antioxidant genes, including *catalase* (*cat*), *superoxide dismutase* (*sod*), *glutathione peroxidase* (*gpx*), and *glutathione reductase* (*gsr*) in the gills, hepatic cecum, and intestines.

**Figure 14:**
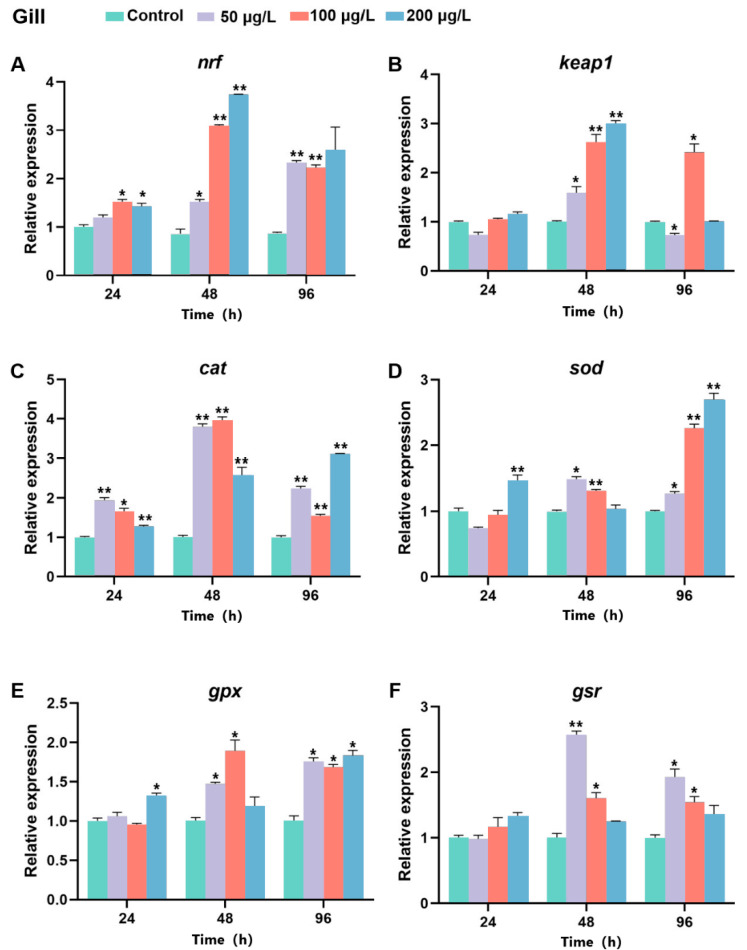
Expression profiles of *Bjnrf*, *Bjkeap*1, and antioxidant-related genes in *B. japonicum* gill exposed to BaP. The expression of *Bjnrf* (**A**), *Bjkeap*1 (**B**), *catalase* (*cat*) (**C**), *superoxide dismutase* (*sod*) (**D**), *glutathione peroxidase* (*gpx*) (**E**), and *glutathione reductase* (*gsr*) (**F**) was detected by qRT-PCR at 24 h, 48 h, and 96 h after 50 μg/L, 100 μg/L, and 200 μg/L BaP exposure. The *β-actin* gene was used as the internal control for normalization. The symbol * indicates *p* < 0.05, ** indicates *p* < 0.01.

In the gill, the expression level of *Bjnrf* showed an increase following exposure to BaP at all concentrations (Figure 14A). Nevertheless, the expression of *Bjkeap*1 exhibited no significant change at 24 h but increased significantly at 48 h. After 96 h, *Bjkeap*1 expression was suppressed in groups exposed to 50 and 200 μg/L of BaP (Figure 14B). The expression levels of antioxidant enzyme genes also showed significant increases, with varying degrees of upregulation across BaP concentrations. After exposure to 50 μg/L or 100 μg/L BaP, the mRNA levels of *cat*, *gpx*, and *gsr* initially rose, peaking at 48 h before declining (Figure 14C,E,F). In contrast, *sod* expression peaked after 96 h at high BaP concentration (200 μg/L) (Figure 14D).

**Figure 15:**
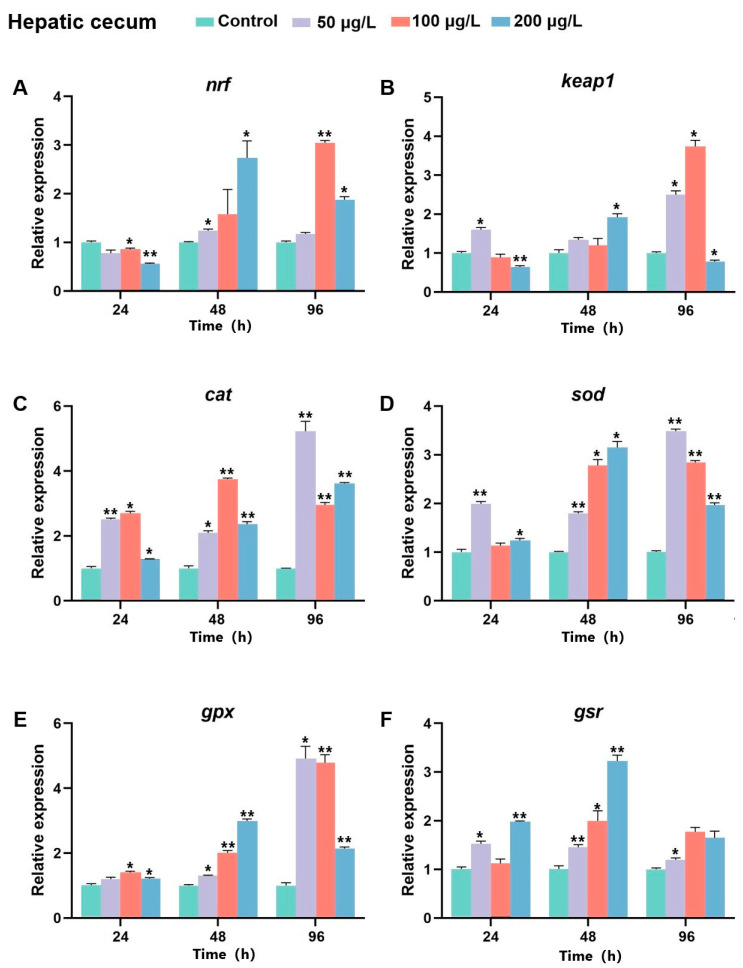
Expression profiles of *Bjnrf*, *Bjkeap*1, and antioxidant-related genes in *B. japonicum* hepatic cecum exposed to BaP. The expression of *Bjnrf* (**A**), *Bjkeap*1 (**B**), *catalase* (*cat*) (**C**), *superoxide dismutase* (*sod*) (**D**), *glutathione peroxidase* (*gpx*) (**E**), and *glutathione reductase* (*gsr*) (**F**) was detected by qRT-PCR at 24 h, 48 h, and 96 h after 50 μg/L, 100 μg/L, and 200 μg/L BaP exposure. The *β-actin* gene was used as the internal control for normalization. The symbol * indicates *p* < 0.05, ** indicates *p* < 0.01.

In the hepatic cecum, the expression of *Bjnrf* exhibited a decreasing trend at 24 h, but subsequently increased at 48 h and 96 h. A substantial elevation in *Bjnrf* transcript levels was observed at 48 h after high-dose (200 μg/L) exposure and at 96 h following moderate-dose (100 μg/L) exposure (Figure 15A). Similarly, *Bjkeap*1 expression also showed a significant increase in these treatment groups, through a notable inverse trend between *Bjnrf* and *Bjkeap*1 expression was observed under prolonged high-dose exposure (Figure 15B). The expression levels of antioxidant genes increased after BaP exposure in the hepatic cecum. The transcripts of *cat*, *sod*, and *gpx* were significantly upregulated at 48 h and 96 h across different BaP concentrations, reaching maximal expression at 96 h after 50 μg/L BaP exposure (Figure 15C–E). However, *gsr* expression peaked at 48 h under high-dose (200 μg/L) exposure, with no significant elevation observed at 96 h after moderate or high-dose exposures (Figure 15F).

**Figure 16:**
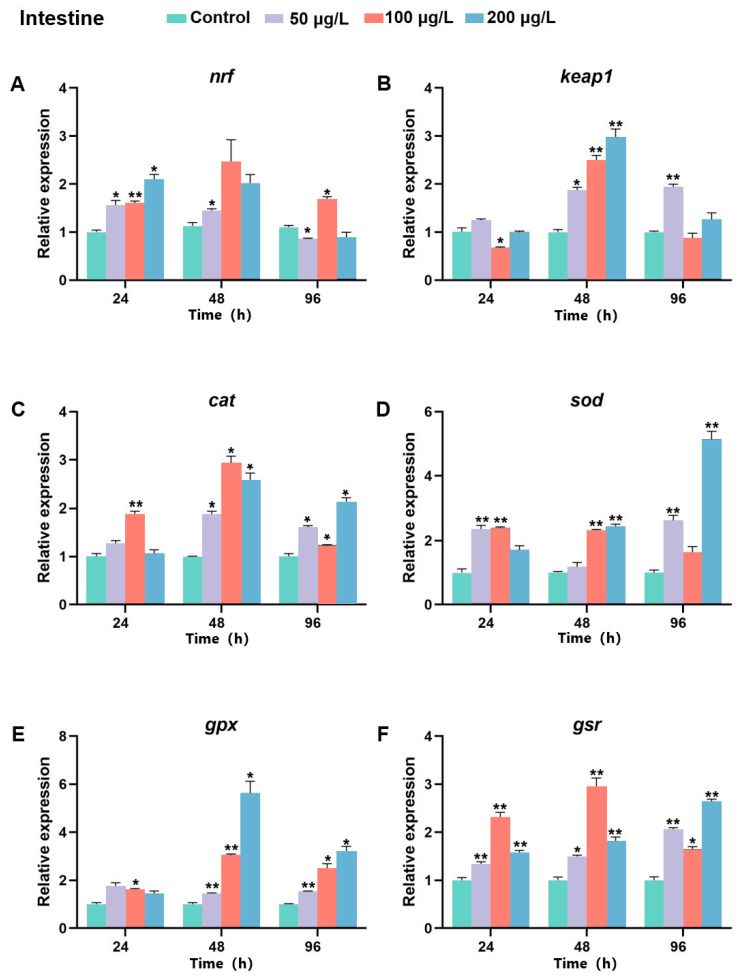
Expression profiles of *Bjnrf*, *Bjkeap*1, and antioxidant-related genes in *B. japonicum* intestine exposed to BaP. The expression of *Bjnrf* (**A**), *Bjkeap*1 (**B**), *catalase* (*cat*) (**C**), *superoxide dismutase* (*sod*) (**D**), *glutathione peroxidase* (*gpx*) (**E**), and *glutathione reductase* (*gsr*) (**F**) was detected by qRT-PCR at 24 h, 48 h, and 96 h after 50 μg/L, 100 μg/L, and 200 μg/L BaP exposure. The *β-actin* gene was used as the internal control for normalization. The symbol * indicates *p* < 0.05, ** indicates *p* < 0.01.

In the intestine, *Bjnrf* mRNA levels generally exhibited an increasing trend in response to BaP, except under prolonged treatment at 50 and 100 μg/L BaP (Figure 16A). However, the expression levels of *Bjkeap*1 were significantly upregulated, particularly at 48 h across BaP concentrations (Figure 16B). The transcripts of antioxidant genes also increased in response to BaP in the intestine. Notably, *cat*, *gpx*, and *gsr* were significantly upregulated at both 48 h and 96 h, with maximal expression occurring at 48 h after moderate and high-dose exposures (Figure 16C,E,F). Meanwhile, the expression level of *sod* was reached its peak following prolonged high-dose exposure (96 h, 200 μg/L Bap) (Figure 16D).

## 3. Discussion

To maintain cellular homeostasis, organisms have evolved various anti-stress systems to cope with xenobiotic and oxidative stress. Among these, the KEAP1-NRF2 pathway in vertebrates plays a crucial role in detoxifying environmental pollutants, resisting oxidative stress, and regulating reactive oxygen species (ROS) metabolism [7,48]. While NRF2 homologues have been identified across a range of species, there are differences in regulatory mechanisms between vertebrates and invertebrates [2]. The amphioxus, an evolutionary node species, presents an intriguing model for studying these mechanisms, though its KEAP1-NRF2 pathway remains poorly understood. In this study, we successfully cloned the ORFs of *nrf* and *keap*1 from *B. japonicum*. The secondary and tertiary structures of the deduced proteins indicated that we cloned the correct target sequences, which we designated as *Bjnrf* and *Bjkeap*1, respectively. Notably, unlike vertebrates, which possess multiple NRF paralogues (e.g., NRF1, NRF2, NRF3, and NF-E2 in mammals), amphioxus species have only a single NRF gene locus. This difference may result from two rounds of whole-genome duplication in the vertebrate lineage [49].

Phylogenetic and detailed domain structure analyses suggested that BjNRF represents an ancestral type, containing the Neh1-7 domains and endoplasmic reticulum (ER)-binding domain. Sequence alignment indicated that while Neh1, Neh2, Neh3, and Neh6 domains are conserved between BjNRF and vertebrate NRF2, the Neh4, Neh5, and Neh7 domains are less so.

The identification of conserved motifs and active sites in the Neh1 and Neh2 domains of BjNRF suggested that its DNA-binding manner, as well as the capability of interaction with KEAP1, may be similar to vertebrate NRF2. The Neh1 domain is involved in heterodimerization with sMaf and binding to ARE sequences, while the Neh2 domain facilitates interaction with KEAP1 through the DLG and ETGE motifs, forming a “hinge and latch” structure with the Kelch domain of KEAP1 [50,51,52]. In particular, the lysine residues between the DLG and ETGE motifs are recognized as a target position for NRF2 ubiquitination [53]. The existence of the four lysine sites in BjNRF suggests that its activity is suspected to be regulated through the ubiquitin proteasome system. Even though NRF1 also possesses the DLG and ETGE motifs, its transcriptional activity is not affected by the mutation of these motifs, implying the different functional contributions of these motifs between vertebrates NRF1 and NRF2 [33]. In comparing the Neh2 domains of NRF1, NRF2, and BjNRF, Neh2 of the BjNRF domain appears more similar to that of NRF2, due to the conserved positioning of lysine sites and the distance between the DLG and ETGE motifs. This distance, highly conserved among vertebrate NRF2 proteins, likely plays a crucial role in maintaining the structural integrity required for effective KEAP1 interaction [2]. It is interesting to note that the DLG-ETGE distance in BjNRF is similar to that of vertebrate NRF2, contrasting with the shorter distance observed in ascidians and the longer distance in fruit flies.

The Neh3/4/5 domains serve as transactivation regions. The Neh3 interacts with CHD6, and the Neh4/5 bind CBP and BRG1 to facilitate gene transcription [36,54]. A conserved VFLVPK motif was identified in the Neh3 domain of BjNRF, whereas homologous motifs in the Neh4/5 domains of NRF2 were incomplete in BjNRF. This observation was found in other examined invertebrates as well, suggesting that the Neh3 domain may be more ancient than Neh4/5 domains. Furthermore, the TRAM motif in the Neh4 domain has been reported to be well conserved in NRF2 but absent in NRF1 [2]. It is noteworthy that although BjNRF also lacked the TRAM motif, such as NRF1, the sequence alignment was shown to be less conserved in this region between BjNRF and NRF1.

The Neh6 domain is essential for the negative regulation of NRF via GSK-3-dependent phosphorylation and subsequent β-TrCP-mediated ubiquitination [38]. However, only the DSGIS motif was found in BjNRF and other examined invertebrate NRFs, suggesting that this motif is evolutionarily ancient and that the phosphorylation-dependent degradation of BjNRF may occur in amphioxus.

Another notable finding is the presence of an NHB domain at the N-terminus of BjNRF, an ER-binding domain unique to NRF1. In vertebrates, the NHB domain anchors NRF1 to the ER, allowing it to respond to ER stress, which differs from NRF2’s cytoplasmic localization and activation mechanism [39,50,55,56]. The presence of this domain in BjNRF raises the possibility that it may similarly respond to ER stress, supporting the idea that BjNRF represents an evolutionary intermediate, consistent with the ancestral NRF model for deuterostomes proposed by Fuse and Kobayashi [2].

In addition to BjNRF, BjKEAP1 has been identified as an ancestral type through phylogenetic and domain structure analyses. Sequence alignment showed that KEAP1 is highly conserved across the investigated species. Notably, BjKEAP1 contained all the conserved domains, including the BTB, IVR, and DGR/Kelch domains, as well as multiple conserved active sites. As a sensor for electrophiles, KEAP1 is rich in cysteine residues, which serve as sensor amino acids due to their high reactivity [57]. Among these residues, Cys-151, Cys-273, and Cys-288 in mouse KEAP1 have been shown to function as electrophile sensors in vivo [43,44,45]. BjKEAP1 was found to possess Cys-273 and Cys-288 residues, consistent with the notion that these are the most evolutionarily ancient sensor residues [2]. Interestingly, while the Cys-151 residue is present in vertebrates and chordates such as ascidians, it is absent in chordate amphioxus, highlighting an evolutionary divergence in electrophile sensing mechanisms.

Vertebrate NRF2 is predominantly expressed in the liver and kidney, organs central to detoxification, as well as in tissues frequently exposed to external environments, such as the skin, lungs, and gastrointestinal tract [58]. In contrast, NRF1 is highly expressed in the heart, muscle, liver, kidney, and secretory compartments, reflecting its broader role in regulating development and various cellular processes beyond antioxidant responses [59]. Similar to vertebrate NRF2, BjNRF exhibited high mRNA expression levels in the gill, hepatic cecum, and intestine, tissues continuously exposed to external environments. A comparable expression pattern was also observed for *Bjkeap*1, suggesting that both genes may play pivotal roles in responding to xenobiotic stimuli and maintaining cellular homeostasis under environmental stress.

As a partner protein of NRF2 and an adaptor for the ubiquitin ligase complex, KEAP1 plays a pivotal role in maintaining NRF2 at low cellular levels through proteasomal degradation [9]. The C-terminal DGR/Kelch domain of KEAP1 is essential for its interaction with NRF2. Subcellular localization studies demonstrate that KEAP1, lacking this domain, is unable to retain NRF2 in the cytoplasm [60]. Consistently, experiments have revealed that when NRF2 is expressed alone in 293T cells, it predominantly localizes to the nucleus. However, co-expression of NRF2 and KEAP1 shifts NRF2 localization primarily to the cytoplasm [61]. These findings align with the observed subcellular localization patterns of BjKEAP1 and BjNRF, suggesting that BjKEAP1 may similarly inhibit BjNRF nuclear translocation by retaining it in the cytoplasm. It indicates that BjNRF is likely regulated by a KEAP1-dependent mechanism akin to that of vertebrate NRF2.

The interaction between NRF2 and KEAP1 can be disrupted by an NRF2 activator, such as sulforaphane (SFN) [62]. SFN, a phytochemical compound from the isothiocyanate family, is naturally found in cruciferous vegetables. As a well-characterized NRF2 activator, SFN modifies the cysteine residues of KEAP1, leading to the dissociation of NRF2 from KEAP1 and its subsequent translocation into the nucleus, where it upregulates downstream phase II detoxification genes [63]. In this study, after treatment with 20 μM SFN, BjNRF translocation was observed in some co-infected HEK293 cells, suggesting that SFN can also influence the interaction between BjNRF and BjKEAP1. Notably, previous studies have identified that Cys151, located in the BTB domain of KEAP1, is the most critical cysteine residue sensitive to sulforaphane [60,64]. However, this residue is absent in amphioxus (Figure 6). Despite this, BjKEAP1 retains five cysteine residues—Cys38, Cys77, Cys226, Cys434, and Cys489—which are also preferentially modified by sulforaphane (Supplementary Figure S7) [65], probably contributing to the disruption of the BjNRF–BjKEAP1 interaction. Nevertheless, further studies are necessary to elucidate the molecular details of this regulatory interaction in amphioxus.

SFN facilitates NRF2 activation not only by promoting its nuclear accumulation but also by enhancing its transcriptional expression [63]. Evidence suggests that SFN treatment induces NRF2 mRNA through epigenetic modifications, including DNA demethylation at the NRF2 promoter and the inhibition of DNA methyltransferases and histone deacetylase [66,67]. Previous studies have reported that SFN elevates the transcriptional levels of NRF2 and its downstream phase II detoxification genes in non-dialysis patients with chronic kidney disease [68], in bovine oocytes exposed to paraquat [69], in prostate cancer TRAMP C1 cells [66], and in fathead minnow epithelial cells (FHM) [70]. In this study, SFN significantly induced *Bjnrf* expression across all investigated tissues, with particularly high fold changes observed in the gill and hepatic cecum. Notably, *Bjnrf* transcription levels returned to baseline after 6 or 12 h of treatment (Figure 11A, Figure 12A and Figure 13A). This result is consistent with observations in fathead minnow epithelial cells treated with SFN, where Nrf2 transcription levels significantly increased between 6 and 12 h before returning to baseline [70]. Additionally, the *Bjkeap*1 transcription level was also upregulated at 6 h. In the gill and hepatic cecum, a significant increase in *Bjkeap*1 mRNA levels was still observed, despite the transcription level of *Bjnrf* returning to baseline (Figure 11B and Figure 12B). Notably, KEAP1 contains an antioxidant response element (ARE) within its promoter region, allowing its upregulation in response to NRF2 activation [71,72]. This autoregulatory feedback loop facilitates NRF2 degradation, preventing excessive accumulation that could otherwise lead to adverse effects, such as apoptosis and tumorigenesis [64].

SFN also induced the expression of phase II detoxification genes in amphioxus, including *gclc*, *gclm*, *prdx*, and *gstp*. Glutathione (GSH) is a crucial antioxidant that mitigates oxidative stress, while glutamate–cysteine ligase (GCL), composed of the GCLC and GCLM subunits, is the rate-limiting enzyme in GSH biosynthesis [73]. Both GCLC and GCLM are directly regulated by NRF2 and serve as robust markers of NRF2 activity [74]. Additionally, glutathione S-transferase P (GSTP), a member of the GST superfamily, facilitates the conjugation of GSH to electrophiles and xenobiotics and is also directly activated by NRF2 [75,76]. In all investigated tissues, SFN significantly upregulated the expression of *gclc*, *gclm*, and *gstp*, highlighting the strong association between GSH-mediated detoxification and BjNRF activation. Peroxiredoxins (PRDX), other phase II detoxification enzymes regulated by NRF2, play a vital role in cellular defense against oxidative stress by neutralizing peroxides and acting as sensors in hydrogen peroxide-mediated signaling pathways [77]. In this study, *prdx* was significantly upregulated at 6 h post SFN treatment, indicating its activation relevant to BjNRF. Overall, the upregulation of these key NRF2 downstream phase II detoxification genes in response to BjNRF activation suggests a conserved NRF2 regulatory mechanism between amphioxus and vertebrates.

Benzo[a]pyrene (BaP) is a widespread marine environmental pollutant that poses a threat to marine ecosystems [78]. Previous studies have reported that BaP-induced oxidative stress can trigger the expression of *nrf*2 [79,80]. As a phase I enzyme ligand, BaP binds to the aryl hydrocarbon receptor (AHR), leading to the generation of endogenous ROS, which subsequently activate the NRF2-KEAP1 system [81]. Additionally, this system can be directly activated through the AHR-XRE (xenobiotic response element) pathway [82]. In this study, exposure to varying concentrations of BaP significantly upregulated *Bjnrf* transcription, particularly after 48 h, across all examined tissues, including the gill, hepatic cecum, and intestine. The elevated mRNA levels suggest that *Bjnrf* is highly responsive to BaP-induced oxidative stress. Similar oxidative stress-mediated increases in *nrf*2 mRNA have been reported in other organisms, such as scallops (*Chlamys farreri*) in response to BaP exposure [83], European eel hepatocytes exposed to H_2_O_2_ [84], Antarctic silverfish embryos subjected to environmental changes [85], and zebrafish embryos in response to organic pollutants [86].

Moreover, *Bjnrf* expression patterns varied among different tissues. In the gill, transcription levels exhibited a consistent upward trend throughout the exposure period. In contrast, in the hepatic cecum, it showed an initial downregulation at 48 h, followed by upregulation, while in the intestine, it displayed a declining trend after prolonged exposure at 96 h. Similar to the expression pattern in the hepatic cecum, the *nrf*2 mRNA level in clam digestive glands also exhibited initial downregulation followed by upregulation when exposed to BaP [80]. Conversely, European eel hepatocytes exposed to H_2_O_2_ showed an initial increase in *nrf*2 levels, followed by downregulation after extended exposure [84], aligning with the observed pattern in the amphioxus intestine. These differential expression patterns suggest that the timing of *Bjnrf* induction and cessation in response to oxidative stress varies across tissues. Such variability likely reflects distinct regulatory mechanisms tailored to tissue-specific functions and oxidative stress thresholds, underscoring the complexity of NRF-mediated stress responses.

The expression of *Bjkeap*1 exhibited either a similar or inverse relationship with *Bjnrf* mRNA levels depending on the exposure period. At 48 h after BaP exposure, *Bjkeap*1 mRNA levels were upregulated across all examined tissues, consistent with the trend of *Bjnrf* expression. This oxidative stress-induced concurrent upregulation of both *nrf*2 and *keap*1 has also been reported in scallop digestive glands exposed to BaP [83], European eel hepatocytes in response to H_2_O_2_ [84], and human keratinocytes exposed to arsenic [87]. Upregulation of KEAP1 through an autoregulatory feedback loop has been reported to prevent excessive NRF2 accumulation, thereby mitigating potential adverse effects [58,71,87]. However, during prolonged BaP exposure, a negative correlation between *Bjnrf* and *Bjkeap*1 expression levels was observed. In the gill, at 96 h post-exposure to 50 or 100 μg/L BaP, *Bjnrf* mRNA levels increased while Bjkeap1 levels either decreased or remained unchanged significantly. A similar pattern was observed in the hepatic cecum at 96 h after exposure to 200 μg/L BaP and in the intestine at 96 h following exposure to 100 μg/L BaP. This downregulation of *Bjkeap*1 expression may reduce BjNRF degradation, thereby extending its activity to counter prolonged oxidative stress. A comparable negative correlation between *nrf*2 and *keap*1 expression was also observed in clam digestive glands under long-term BaP exposure [80]. These findings suggest that while the BjKEAP1-BjNRF regulatory feedback mechanism maintains oxidative stress homeostasis during acute stress, prolonged exposure may attenuate *Bjkeap*1 expression to enhance the protective activity of *Bjnrf*, potentially reflecting a finely tuned adaptive response to sustained environmental stressors.

The enzymes superoxide dismutase (SOD), catalase (CAT), glutathione peroxidase (GPx), and glutathione reductase (GSR) play pivotal roles in mitigating oxidative stress and are widely recognized as key antioxidant biomarkers in marine organisms [88,89]. SOD catalyzes the dismutation of superoxide anions into hydrogen peroxide (H_2_O_2_), which is subsequently reduced by CAT and GPx. Meanwhile, GSR regenerates reduced glutathione from its oxidized form, ensuring intracellular redox balance [89]. Studies have shown that mammalian CAT and GPx possess antioxidant response elements (AREs) [90], as well as SOD in fish [91], suggesting that these genes may be potential downstream targets of BjNRF. In the present study, significant upregulation of antioxidant genes was observed primarily at 48 and 96 h post exposure to BaP, highlighting the activation of the antioxidant defense system. Furthermore, the expression trends of *cat* and *gpx* seemed to correlate with changes in *Bjnrf* expression upon exposure to BaP, indicating an ARE-dependent activation mechanism for these genes. This finding aligns with previous reports of consistent expression patterns among *cat*, *gpx*, and *nrf*2 in scallops [83], European eels [84], and carp [91]. Conversely, the expression profiles of *sod* and *gsr* did not correlate with *Bjnrf* expression. For instance, in the intestine, under prolonged high-dose BaP exposure, both genes continued to exhibit an upward trend despite the repression of *Bjnrf*. This discrepancy suggests that the regulation of *sod* and *gsr* may involve additional transcriptional mechanisms independent of *Bjnrf*.

## 4. Materials and Methods

### 4.1. Animal and Cell Culture

Adult *B. japonicum* were cultured in aerated fresh seawater and fed single-celled algae twice daily. All animal experiments in this study were approved by the Ethical Committee of Ocean University of China (OUC-AE-2024-046). HEK-293T cells were cultured at 37 °C with 5% CO_2_ in Dulbecco’s modified Eagle medium (DMEM, Gibco, Shanghai, China) supplemented with 10% (*v*/*v*) fetal bovine serum (FBS) and 1% penicillin-streptomycin, as described previously [92].

### 4.2. Cloning and Sequencing of B. japonicum nrf and keap1 cDNAs

Total RNA from *B. japonicum* was extracted using RNAiso plus (Takara Biotechnology, Dalian, China) and purified with the Total RNA Kit I (Omega Bio-Tek, Guangzhou, China), following the manufacturer’s instructions. Reverse transcription was performed using the Hiscript III RT SuperMix (+gDNA wiper) Kit (Vazyme, Nanjing, China) according to the manufacturer’s instructions.

To amplify the ORFs of *B. japonicum nrf* and *keap*1 genes, PCR was performed with primer pairs P1/P2 and P3/P4 (see Supplementary Table S1). Specific primers were designed based on the predicted gene sequences from the *B. japonicum* transcriptome database (unpublished data). PCR products were purified with a Gel Extraction Kit (Omega Bio-Tek, Norcross, GA, USA), subcloned into a PGEM^®^-T vector (Promega, Madison, WI, USA), and then transformed into Trans 1T-1 *E. coli* (TransGen Biotech, Beijing, China), according to the manufacturer’s instructions. The amplified DNA fragments were verified by sequencing.

### 4.3. Sequence Analysis

Homology searches in the non-redundant protein database (nr) were conducted using the BLAST online server (https://blast.ncbi.nlm.nih.gov, accessed on 23 September 2023). Protein sequence alignment was performed using the MUSCLE method in the MegAlign program of the DNASTAR 7.1 software package. Phylogenetic trees were constructed based on amino acid sequence alignments using the Maximum Likelihood (ML) and the Neighbor-Joining (NJ) algorithms in the MEGA 7.0 software, with confidence levels of individual nodes assessed by bootstrap analysis (1000 resampling). Domain predictions were performed using the SMART program (http://smart.embl-heidelberg.de/, accessed on 23 September 2023). Protein 3D structures were predicted with Alphafold2 and visualized with PyMOL 3.0 software.

### 4.4. In Situ Hybridization

All reagents for in situ hybridization were prepared with sterilized water treated with 0.1% (*v*/*v*) diethyl pyrocarbonate (DEPC, Sangon Biotech, Shanghai, China). Amphioxi were fixed in 4% (*w*/*v*) paraformaldehyde in 100 mM PBS (pH 7.4) at 4 °C for 12 h. The specimens were then segmented into three pieces, followed by sequential washing, dehydration, clearing, wax impregnation, and paraffin embedding. Thin sections were then sliced, mounted, and baked to produce paraffin-embedded tissue sections. Sections were deparaffinized in xylene, rehydrated, and equilibrated in double-distilled water, then hybridized with DIG-labeled antisense RNA probes at 50 °C for 12 h. Control experiments were performed with sense RNA probes. DIG-labeled *Bjnrf* and *Bjkeap*1-specific RNA probes were synthesized by in vitro transcription using a DIG RNA labeling kit (Roche, Sigma-Aldrich, Shanghai, China), with primers P9, P10, P11, and P12 (see Supplementary Table S1). Sections were preincubated with 1% blocking solution (Roche, Basel, Switzerland) for 2 h at room temperature, then incubated with 0.1% DIG antibody in blocking solution at 4 °C for 12 h. After washing and staining, images were captured using a BX51 Olympus microscope (Olympus, Tokyo, Japan).

### 4.5. Plasmid Construction

Expression vectors were constructed using the homologous recombination method. Specific primers for *Bjnrf* and *Bjkeap*1 were designed using the online program Primer Design for ClonExpress Entry, with overlapping sequences from pcDNA3.1 (Amp^+^) vectors containing fluorescent protein (Beyotime, Shanghai, China). Coding sequences of *Bjnrf* and *Bjkeap*1 were PCR-amplified using primer pairs P5/P6 and P7/P8 (Supplementary Table S1), respectively, then recombined into the NotI-digested pcDNA3.1-EGFP or EcoRI-digested pcDNA3.1-mCherry using ClonExpress II One Step Cloning Kit (Vazyme, Nanjing, China). The resulting expression vectors, pcDNA3.1-Bjnrf-EGFP and pcDNA3.1-Bjkeap1-mCherry, were verified by sequencing.

### 4.6. Subcellular Localization

Recombinant plasmids (pcDNA3.1-Bjnrf-EGFP or pcDNA3.1-Bjkeap1-mCherry) were transfected into the HEK-293 cells using Hieff Trans™ Liposomal Transfection Reagent (Yeasen, Shanghai, China), following the manufacturer’s instructions. After 36~48 h of transfection, cells were fixed with 4% (*v*/*v*) paraformaldehyde (PFA) for 20 min, and permeabilized with 0.1% Triton X-100. Nuclear DNA was stained in a solution containing 0.1% (*v*/*v*) DAPI (4′,6-diamidino-2-phenylindole). Confocal images were obtained using the LAS X laser scanning microscope system.

Sulforaphane (SFN; CAS: 4478-93-7; MCE, US; MW: 177.29) was dissolved in dimethyl sulfoxide (DMSO; Solarbio, Beijing, China; purity > 99.9%) to prepare a stock solution.

After 18–24 h of co-transfection with the recombinant plasmids pcDNA3.1-Bjnrf-EGFP and pcDNA3.1-Bjkeap1-mCherry, HEK-293 cells were treated with 20 μM SFN for 6 h. The control groups were exposed to an equivalent concentration (0.1%) of DMSO in culture medium. Nuclear DNA staining, cell fixation, and confocal imaging were performed as described above.

### 4.7. SFN Treatment and Sample Collection

SFN stock solution was prepared as described above and added to filtered seawater to achieve nominal SFN concentrations of 50 μM. The control groups were treated with an equal concentration (0.1%) of DMSO in seawater. During SFN exposure, no feeding was provided. Amphioxi were randomly divided into two groups of 25 individuals each, and samples of 5 individuals per group were collected at 6, 12, 24, 48, and 96 h post-exposure. Hepatic cecum, gills, and intestines were collected, preserved in RNAiso Plus (Takara, Dalian, China), and stored at −80 °C.

### 4.8. BaP Exposure and Sample Collection

Benzo[a]pyrene, BaP (CAS:50-32-8; J&K, Beijing, China; MW: 252.31) was dissolved in dimethyl sulfoxide (DMSO, Solarbio, Beijing, China, purity > 99.9%) to create a stock solution. This solution was added to filtered seawater to achieve nominal BaP concentrations of 10, 50, 100, and 200 μg/L. The control groups were treated with an equal concentration (0.1%) of DMSO in seawater. During the Bap exposure experiment, no feeding was provided. Amphioxi were randomly divided into 5 groups of 30 individuals each, and samples of 10 individuals per group were collected at 24, 48, and 96 h post exposure. Hepatic cecum, gills, and intestines were collected, preserved in RNAiso Plus, and stored at −80 °C.

### 4.9. qRT-PCR

Total RNA extraction, purification, and cDNA synthesis were performed as previously described. The mRNA expression of *Bjnrf*, *Bjkeap*1, *gclm*, *prdx*, *gclc*, *gstp*, *sod*, *cat*, *gpx*, and *gsr* was detected by qRT-PCR with specific primer pairs (Supplementary Table S1). *β-actin* was used as an internal reference gene. qRT-PCR was conducted using the Applied Biosystems™ 7500 system (Thermo Scientific, Shanghai, China), following the manufacturer’s instructions (Vazyme, Nanjing, China). Relative mRNA expression levels were determined using the 2^−ΔΔ^CT method.

### 4.10. Statistics Analysis

Statistical analysis was conducted using GraphPad Prism 9.0. All data represent means of three independent measurements per sample. Experimental data were analyzed by one-way analysis of variance (ANOVA) followed by Dunnett’s *t*-test for group comparisons. Significance was determined at *p* < 0.05 (*), *p* < 0.01 (**), and *p* < 0.001 (***), with values represented as mean ± standard error of the mean (SEM) (*n* = 3).

## 5. Conclusions

This study identifies BjNRF and BjKEAP1 as ancestral forms present in the basal chordate amphioxus. Notably, BjNRF appears to be an evolutionary intermediate, incorporating characteristic domains and motifs from both vertebrates NRF1 and NRF2. Furthermore, this study demonstrates the presence of a functional KEAP1-NRF pathway in amphioxus by illustrating the interaction between BjNRF and BjKEAP1 and their coordinated response to BaP-induced oxidative stress. Additionally, the upregulation of *Bjnrf* and phase II detoxification genes by the NRF2 activator sulforaphane suggests a conserved NRF regulatory mechanism between amphioxus and vertebrates. This work addresses a critical gap in our understanding of the KEAP1-NRF pathway in chordates, shedding light on the evolutionary and functional aspects of stress-response mechanisms in amphioxus. Moreover, with the increasing threat of marine pollution, the conservation of amphioxus faces growing challenges. As a living fossil, this species is experiencing a rapid population decline. The insights gained from this study also provide valuable information for the conservation of amphioxus in deteriorating marine environments.

## Data Availability

Data will be made available on request.

## Supplementary Materials

The following supporting information can be downloaded at: https://www.mdpi.com/article/10.3390/ijms26073427/s1.
