# Supplementary material for: The Ancestral KEAP1-NRF Pathway in Amphioxus Branchiostoma japonicum: Implications for the Evolution of Antioxidant Defense System
Source: Int J Mol Sci. 2025 Apr 6;26(7):3427. doi: 10.3390/ijms26073427 (PMC11989980; doi:10.3390/ijms26073427)
Supplement: Supplementary file 1 [file ijms-26-03427-s001.zip › ijms-3397565-supplementary.pdf]

***Supplementary information for***

# The ancestral KEAP1-NRF pathway in amphioxus *Branchiostoma japonicum*: implications for the evolution of antioxidant defense system

Weichen Li[1, †], Xiaoqian Liang[1, †], Keyu Xiang[1], Hongyan Li[1,2], Yu Zhang[1,2,*]

[1] *College of Marine Life Sciences, Key Laboratory of Evolution & Marine Biodiversity (Ministry of Education) and Institute of Evolution & Marine Biodiversity, Ocean University of China, Qingdao 266003, China*

[2] *Laboratory for Marine Biology and Biotechnology, Qingdao Marine Science and Technology Center, Qingdao 266237, China*

[*] Correspondence: yuzhang@ouc.edu.cn (Y.Z.)

[†] These authors contributed equally to this work.

**Table S1. Primers used in this study**

| Primer name | Sequence (5′– 3′) |
|---|---|
| P1 | ATGGTGAAGAAACATTTCTACGATGG |
| P2 | CTTGCTGTTCTTTGACCCTTTTC |
| P3 | ATGGCGGAAGACGAGCCC |
| P4 | TGAGGGGCCTTTGGTGCA |
| P5 | atatccagcacagtggcggccgcATGGTGAAGAAACATTTCTACGATGG |
| P6 | gccctctagactcgagcggccgcCTTGCTGTTCTTTGACCCTTTTC |
| P7 | tagtccagtgtggtggaattcATGGCGGAAGACGAGCCC |
| P8 | tgctggatatctgcagaattcTGAGGGGCCTTTGGTGCA |
| P9 | TTCAGTTTGCTTGGGACGG |
| P10 | TGGAAGGCTGTTGAGGTGC |
| P11 | TGCTCCGAGAACACCACC |
| P12 | CAGGCAGATAGACTCTTCTTGAT |
| *β-actin*-S | TTCCAGCCTTCATTCCTCG |
| *β-actin*-AS | CGGTGTTGGCGTACAGGTC |
| RT-*Bjnrf*-S | CGGTAATGCCCAGAACCTGA |
| RT-*Bjnrf*-AS | CTCGGACAGGGAGGATGTTT |
| RT-*Bjkeap1*-S | TGCGTCGGTGATGTTCCA |
| RT-*Bjkeap1*-AS | AGTTGGCAAGCGGATAGCA |
| RT-*gclm*-S | GTTGTATGATGTGGAGCGTGTTT |
| RT-*gclm*-AS | GGTTGATAGTAGGCTTGACTCTGG |
| RT-*prdx*-S | GACTGCTGTAGTTGGGAATGGG |
| RT-*prdx*-AS | CAGTCGGACATACAAAGGTGAAAT |
| RT-*gclc*-S | CCAAAACTTCGTTCCCCTTCA |
| RT-*gclc*-AS | CCAGCAACTTATCAACATCTACCG |
| RT-*gstp*-S | AGGCGGAGAAGGGTTTGTGA |
| RT-*gstp*-AS | GAGGGGAATGGAGTCGTTGG |
| RT-*cat*-S | CATGTTTTGGGACTTCATCTCGC |
| RT-*cat*-AS | ACACGGCTTCGTTGTTCTTG |
| RT-*gpx*-S | TCCCGTGTTCGCCTACCT |
| RT-*gpx*-AS | TTGATTGGCTGCCCGTCT |
| RT-*sod*-S | TATTCGCCCGGCCATTGTAG |
| RT-*sod*-AS | ACTGCTGAGTCTGAGTTCGC |
| RT-*gsr*-S | TTTAACGGCGAGGACACCAA |
| RT-*gsr*-AS | TGGTACATGGGGGTGAAACG |

**Table S2. Sequences used in the alignment and phylogenetic analysis of NRF homologs**

| Protein name | Protein Definition | Accession number |
|---|---|---|
| *Homo sapiens* Nrf1 | endoplasmic reticulum membrane sensor NFE2L1 isoform 1 | NP_003195.1 |
| *Homo sapiens* Nrf2 | nuclear factor erythroid 2-related factor 2 isoform 1 | NP_006155.2 |
| *Homo sapiens* Nrf3 | nuclear factor erythroid 2-related factor 3 | NP_004280.5 |
| *Homo sapiens* Nfe2 | transcription factor NF-E2 45 kDa subunit isoform 1 | NP_001129495.1 |
| *Mus musculus* Nrf1 | endoplasmic reticulum membrane sensor NFE2L1 isoform 1 | NP_032712.2 |
| *Mus musculus* Nrf2 | nuclear factor erythroid 2-related factor 2 | NP_035032.1 |
| *Mus musculus* Nrf3 | nuclear factor erythroid 2-related factor 3 | NP_035033.1 |
| *Mus musculus* Nfe2 | transcription factor NF-E2 45 kDa subunit | NP_032711.2 |
| *Rattus norvegicus* Nrf1 | endoplasmic reticulum membrane sensor NFE2L1 1 | NP_001101763.2 |
| *Rattus norvegicus* Nrf2 | nuclear factor erythroid 2-related factor 2 isoform 1 | NP_001386102.1 |
| *Rattus norvegicus* Nrf3 | nuclear factor erythroid 2-related factor 3 precursor | NP_001291976.1 |
| *Rattus norvegicus* Nfe2 | transcription factor NF-E2 45 kDa subunit | NP_001012224.1 |
| *Bos taurus* Nrf1 | endoplasmic reticulum membrane sensor NFE2L1 isoform X1 | XP_005220647.1 |
| *Bos taurus* Nrf2 | nuclear factor erythroid 2-related factor 2 | NP_001011678.2 |
| *Bos taurus* Nrf3 | nuclear factor erythroid 2-related factor 3 | NP_001071367.2 |
| *Bos taurus* Nfe2 | transcription factor NF-E2 45 kDa subunit isoform X1 | XP_024847435.1 |
| *Pan troglodytes* Nrf1 | endoplasmic reticulum membrane sensor NFE2L1 isoform X1 | XP_001173202.3 |
| *Pan troglodytes* Nrf2 | nuclear factor erythroid 2-related factor 2 isoform X1 | XP_001145876.4 |
| *Pan troglodytes* Nrf3 | nuclear factor erythroid 2-related factor 3 isoform X1 | XP_519002.2 |
| *Pan troglodytes* Nfe2 | transcription factor NF-E2 45 kDa subunit | XP_001170837.2 |
| *Apus apus* Nrf1 | endoplasmic reticulum membrane sensor NFE2L1 | XP_051496341.1 |
| *Apus apus* Nrf2 | nuclear factor erythroid 2-related factor 2 isoform X1 | XP_051480152.1 |
| *Apus apus* Nrf3 | nuclear factor erythroid 2-related factor 3 isoform X1 | XP_051467028.1 |
| *Gallus gallus* | endoplasmic reticulum membrane sensor NFE2L1 | NP_001025927.1 |
| | nuclear factor erythroid 2-related factor 2 | NP_990448.1 |
| *Anas platyrhynchos* | endoplasmic reticulum membrane sensor NFE2L1 isoform X1 | XP_038024868.1 |
| | nuclear factor erythroid 2-related factor 2 | NP_001297706.1 |
| *Chrysemys picta bellii* | endoplasmic reticulum membrane sensor NFE2L1 isoform X1 | XP_005301942.1 |
| | nuclear factor erythroid 2-related factor 2 isoform X1 | XP_005300513.1 |
| | transcription factor NF-E2 45 kDa subunit isoform X1 | XP_023965633.1 |
| | nuclear factor erythroid 2-related factor 3 | XP_005290938.1 |
| *Pogona vitticeps* | nuclear factor erythroid 2-related factor 1 isoform X1 | XP_020641136.1 |
| | nuclear factor erythroid 2-related factor 2 isoform X1 | XP_020661912.1 |
| | transcription factor NF-E2 45 kDa subunit | XP_020640276.1 |
| *Python bivittatus* | endoplasmic reticulum membrane sensor NFE2L1 isoform X1 | XP_007420197.1 |
| | nuclear factor erythroid 2-related factor 2 isoform X1 | XP_025020101.1 |
| | nuclear factor erythroid 2-related factor 3 | XP_025023498.1 |
| | transcription factor NF-E2 45 kDa subunit isoform X1 | XP_025031383.1 |
| *Microcaecilia unicolor* | endoplasmic reticulum membrane sensor NFE2L1 | XP_030077311.1 |
| | nuclear factor erythroid 2-related factor 2 | XP_030066709.1 |
| | nuclear factor erythroid 2-related factor 3 isoform X1 | XP_030058014.1 |
| | transcription factor NF-E2 45 kDa subunit | XP_030054184.1 |
| *Xenopus tropicalis* | endoplasmic reticulum membrane sensor NFE2L1 isoform X1 | XP_002938061.3 |
| | nuclear factor erythroid 2-related factor 2 | NP_001007490.1 |
| | nuclear factor erythroid 2-related factor 3 isoform X1 | XP_002933432.2 |
| | transcription factor NF-E2 45 kDa subunit isoform X1 | XP_012811975.1 |
| *Danio rerio* | endoplasmic reticulum membrane sensor NFE2L1a | NP_998020.2 |

| | | |
|---|---|---|
| | endoplasmic reticulum membrane sensor NFE2L1b | NP_001265771.1 |
| | nuclear factor erythroid 2-related factor 2a | NP_878309.1 |
| *Protopterus annectens* | transcription factor NF-E2 45 kDa subunit isoform X1 | XP_005162426.1 |
| | endoplasmic reticulum membrane sensor NFE2L1 isoform X1 | XP_043911244.1 |
| | nuclear factor erythroid 2-related factor 2 | XP_043931386.1 |
| | transcription factor NF-E2 45 kDa subunit | XP_043939890.1 |
| *Scyliorhinus canicula* | endoplasmic reticulum membrane sensor NFE2L1b isoform X1 | XP_038635591.1 |
| | nuclear factor erythroid 2-related factor 2a | XP_038645249.1 |
| | transcription factor NF-E2 45 kDa subunit-like | XP_038644700.1 |
| *Petromyzon marinus* | endoplasmic reticulum membrane sensor NFE2L1-like isoform X1 | XP_032821486.1 |
| | nuclear factor erythroid 2-related factor 2-like isoform X1 | XP_032801521.1 |
| *Branchiostoma floridae* | endoplasmic reticulum membrane sensor NFE2L1-like isoform X1 | XP_035657503.1 |
| *Branchiostoma belcheri* | PREDICTED: nuclear factor erythroid 2-related factor 1-like isoform X1 | XP_019632182.1 |
| *Ciona intestinalis* | transcription factor protein isoform X1 | XP_009862108.1 |
| *Strongylocentrotus purpuratus* | nuclear factor, erythroid derived 2 isoform X2 | XP_030855722.1 |
| *Limulus polyphemus* | nuclear factor erythroid 2-related factor 1-like | XP_022248403.1 |
| | nuclear factor erythroid 2-related factor 2-like isoform X2 | XP_013772891.1 |
| | segmentation protein cap'n'collar-like | XP_013789222.1 |
| *Penaeus vannamei* | segmentation protein cap'n'collar-like isoform X1 | XP_027222869.1 |
| *Drosophila melanogaster* | cap-n-collar, isoform C | NP_732833.1 |
| *Crassostrea gigas* | nuclear factor erythroid 2-related factor 2 isoform X3 | XP_034317439.1 |
| | endoplasmic reticulum membrane sensor NFE2L1 isoform X2 | XP_034317432.1 |
| *Mizuhopecten yessoensis* | nuclear factor erythroid 2-related factor 2-like isoform X2 | XP_021348785.1 |
| | nuclear factor erythroid 2-related factor 1-like isoform X1 | XP_021348784.1 |
| *Ruditapes philippinarum* | nuclear factor erythroid 2-related factor 2 | AWV55267 |

**Table S3. Sequences used in the alignment and phylogenetic analysis of KEAP1 homologs**

| Species | Protein name | Accession number |
|---|---|---|
| *Homo sapiens* | kelch-like ECH-associated protein 1 | NP_987096.1 |
| *Rattus norvegicus* | kelch-like ECH-associated protein 1 | NP_476493.2 |
| *Mus musculus* | kelch-like ECH-associated protein 1 | NP_001103777.1 |
| *Bos taurus* | kelch-like ECH-associated protein 1 | NP_001094612.1 |
| *Pan troglodytes* | kelch-like ECH-associated protein 1 | NP_001266890.1 |
| *Cuculus canorus* | kelch-like ECH-associated protein 1 | XP_053907779.1 |
| *Vidua chalybeata* | kelch-like ECH-associated protein 1 | XP_053789008.1 |
| *Chrysemys picta* | kelch-like ECH-associated protein 1 | XP_005279608.1 |
| *Pogona vitticeps* | kelch-like ECH-associated protein 1 | XP_020654350.1 |
| *Xenopus tropicalis* | kelch-like ECH-associated protein 1 | NP_001008024.1 |
| *Python bivittatus* | kelch-like ECH-associated protein 1 | XP_007426630.1 |
| *Microcaecilia unicolor* | kelch-like ECH-associated protein 1 | XP_030052921.1 |
| *Danio rerio* | kelch-like ECH-associated protein 1A | NP_878284.2 |
| | kelch-like ECH-associated protein 1B | NP_001106948.1 |
| *Larimichthys crocea* | L.crocea_KEAP1B | XP_019129802.1 |
| | L.crocea_KEAP1A | XP_010738772.1 |
| *Lates calcarifer* | L.calcarifer_KEAP1B | XP_018520553.1 |
| | L.calcarifer_KEAP1A | XP_018519198.1 |
| *Protopterus annectens* | Pa_KEAP1 | XP_043940496.1 |
| *Petromyzon marinus* | Pm_ KEAP1 | XP_032812622.1 |
| *Branchiostoma floridae* | Bf_ KEAP1 | XP_035699363.1 |
| *Branchiostoma belcheri* | Bb_ KEAP1 | XP_019642185.1 |
| *Ciona intestinalis* | Ci_ KEAP1 | XP_002128055.1 |
| *Strongylocentrotus purpuratus* | Sp_ KEAP1 | XP_003724241.1 |
| *Holothuria leucospilota* | Hl_ KEAP1 | KAJ8040271.1 |
| *Limulus polyphemus* | Lp_ KEAP1 | XP_013773777.1 |
| *Hylaeus volcanicus* | Hv_ KEAP1 | XP_053985695.1 |
| *Drosophila melanogaster* | Dm_ KEAP1, isoform B | NP_732202.2 |
| *Crassostrea gigas* | Cg_ KEAP1 | XP_034312567.1 |
| *Mizuhopecten yessoensis* | My_ KEAP1 | XP_021343699.1 |

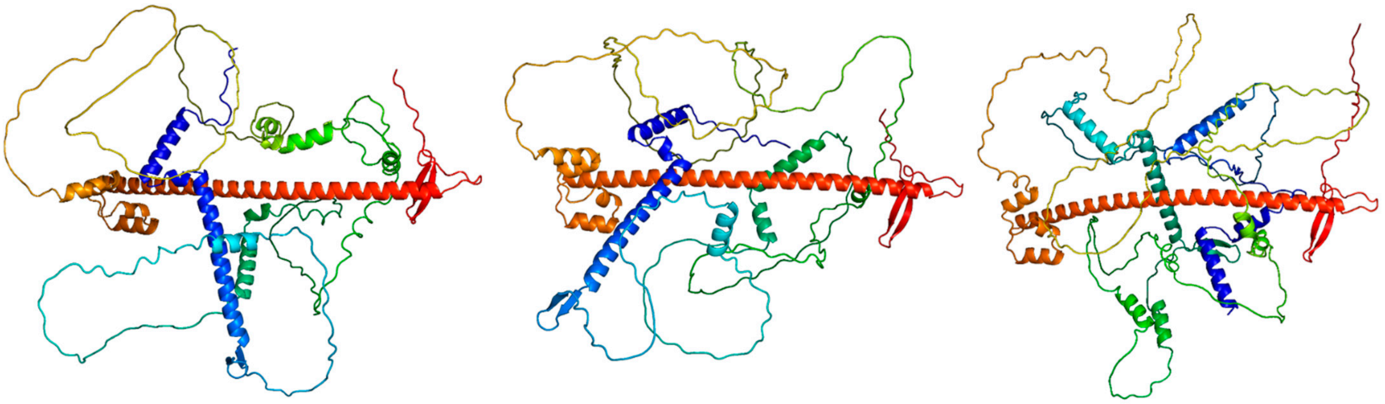

***H. sapiens* NRF2** **A** | ***D. rerio* NRF2a** **B** | ***B. japonicum* NRF** **C**

Figure S1. The three-dimensional structure of NRF2 homologs. The 3D structure of protein was predicted by AlphaFold2 and displayed by Pymol software. (A) Human (*Homo sapiens*) NRF2; (B) Zebrafish (*Danio rerio*) NRF2a; (C) Amphioxus (*Branchiostoma japonicum*) NRF.

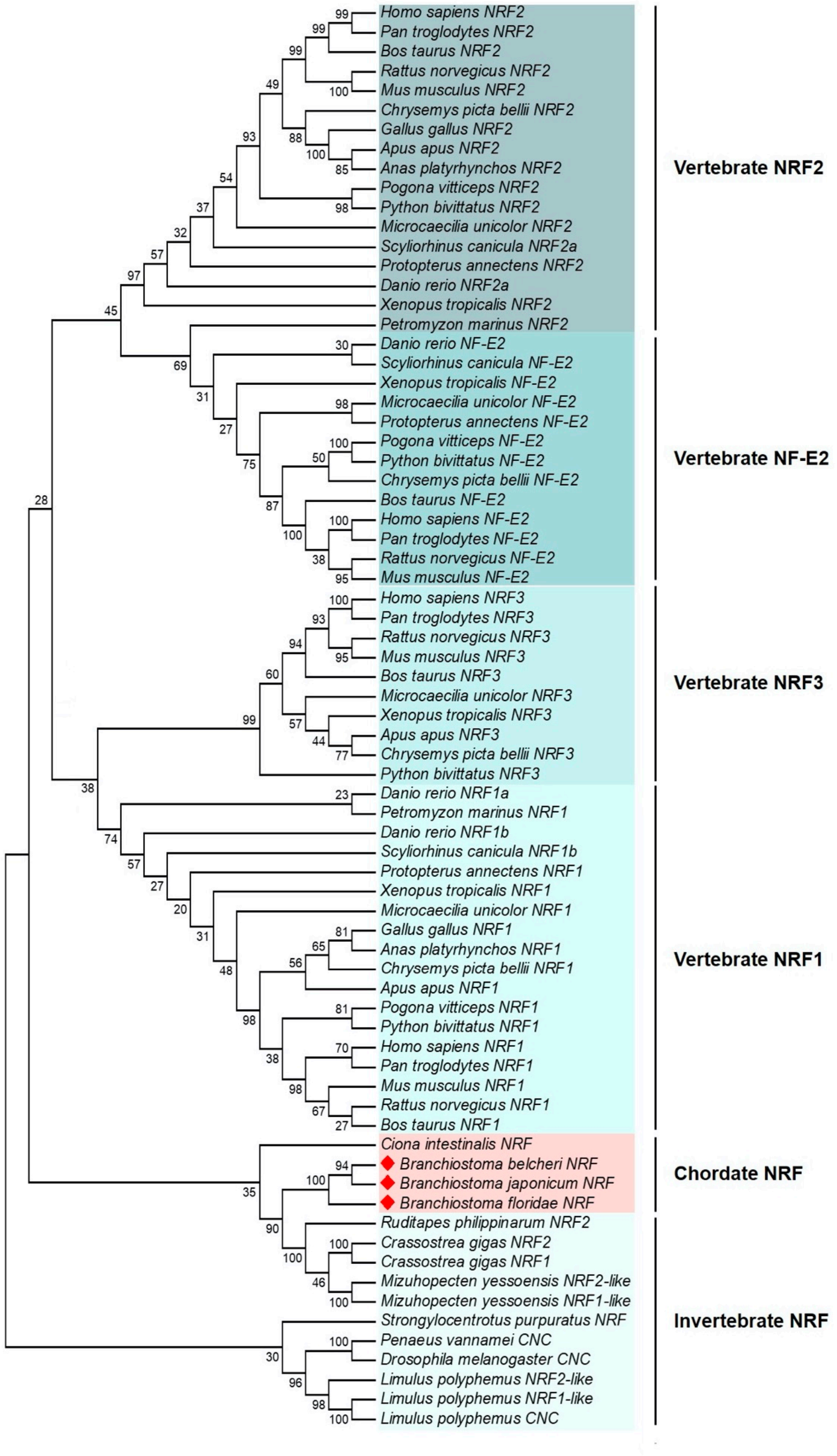


Figure S2. Phylogenetic tree of NRF homologues. The phylogenetic tree was constructed by MEGA 7.0 using the amino acid-based Neighbor-Joining (NJ) algorithm. The reliability of each node was estimated by bootstrapping with 1000 replications. Amphioxus NRF are shown in red background. Accession numbers for sequences used are listed in Table S2 of supplementary data.

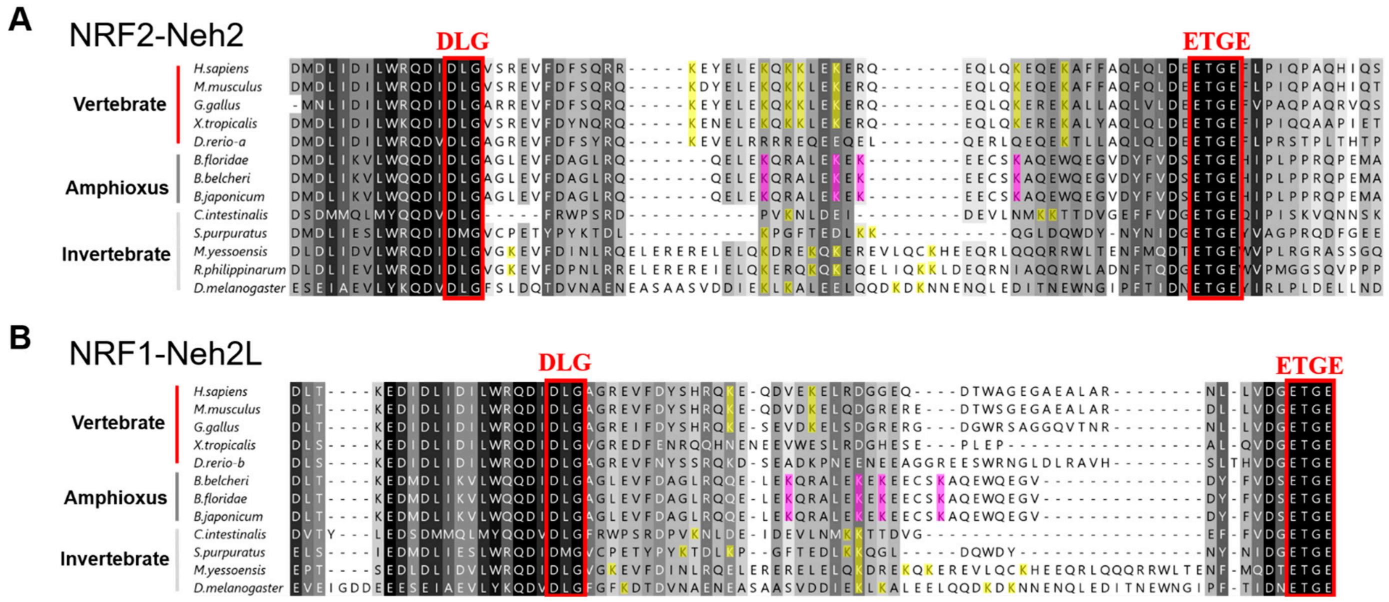


Figure S3. Comparation of domain structure and lysine sites of NRF2 Neh2 and NRF1 Neh2L between amphioxus and various species. Sequence alignment of NRF2 Neh2 domain (A) and NRF1 Neh2L domain (B) among human (*H. sapiens*), mouse (*M. musculus*), chicken (*G. gallus*), frog (*X. tropicalis*), zebrafish (*D. rerio*), amphioxus (*B. floridae*, *B. belcheri,* and *B. japonicum*), ascidian (*C. intestinalis*), sea urchin (*S. purpuratus*), scallop (*M. yessoensis*), clam (*R. philippinarum*), and fruitfly (*D. melanogaster*). The DLG motif and ETGE motif are indicated in red boxes. Between the two motifs, lysine sites of amphioxus and other species are shaded in pink and yellow, respectively.

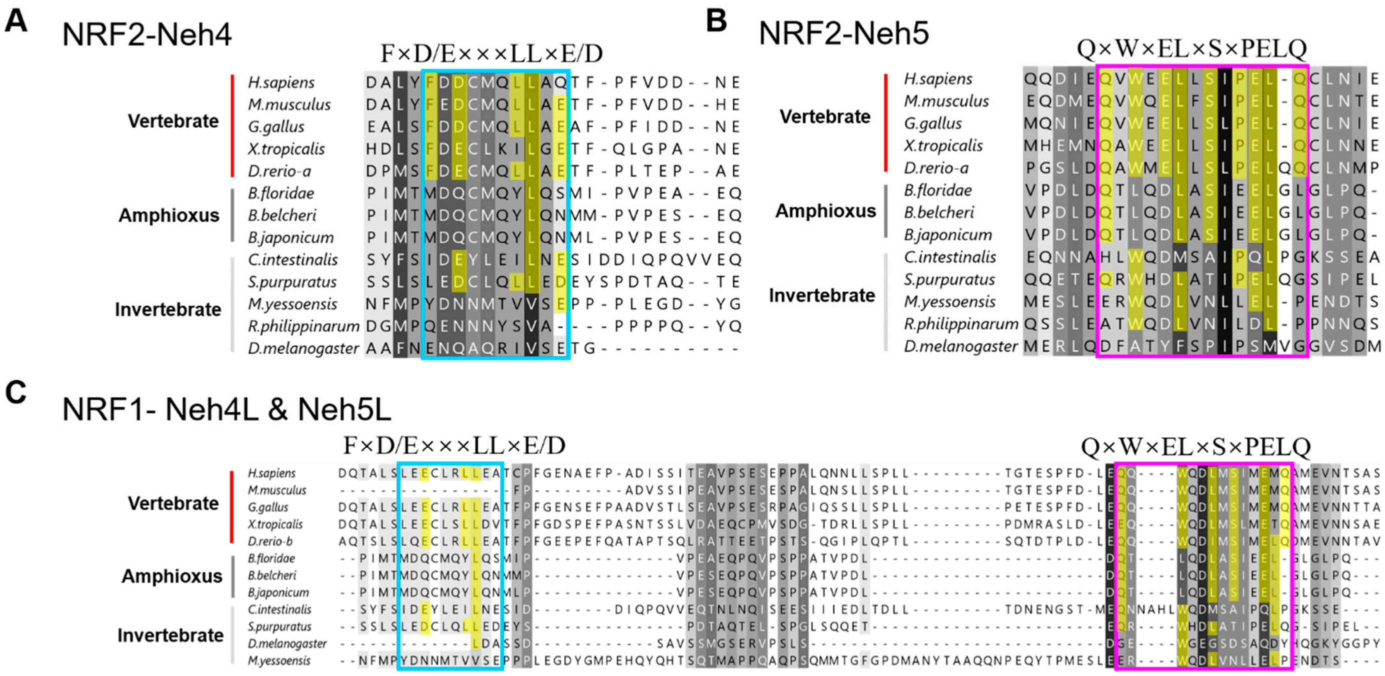


Figure S4. Comparation of NRF2 Neh4/5 and NRF1 Neh4/5L domain structure between amphioxus and various species. Sequence alignment of NRF2 Neh4 (A) and Neh5 domain (B), as well as NRF1 Neh4L/5L domains (C) among human (*H. sapiens*), mouse (*M. musculus*), chicken (*G. gallus*), frog (*X. tropicalis*), zebrafish (*D. rerio*), amphioxus (*B. floridae*, *B. belcheri,* and *B. japonicum*), ascidian (*C. intestinalis*), sea urchin (*S. purpuratus*), scallop (*M. yessoensis*), clam (*R. philippinarum*), and fruitfly (*D. melanogaster*). The TRAM (FxD/ExxxL) motif and the QxWxELxxSxPELQ motif are indicated in red boxes and the basic amino acid residues within these motifs are shaded in yellow.

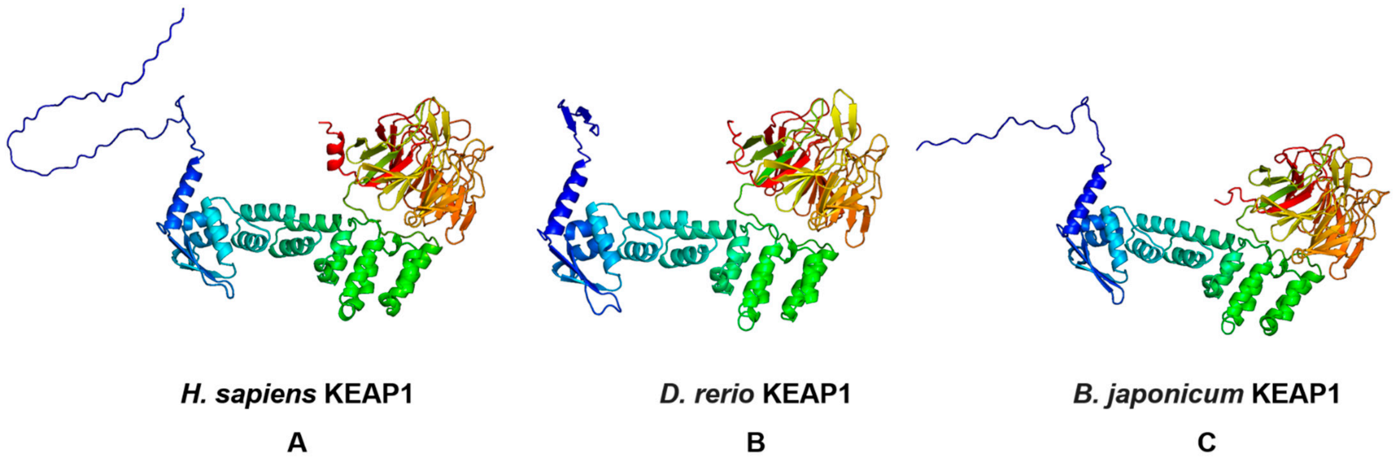


Figure S5. The three-dimensional structure of KEAP1. The 3D structure of KEAP1 was predicted by AlphaFold2 and displayed by Pymol software. (A) Human (*Homo sapiens*) KEAP1; (B) Zebrafish (*Danio rerio*) KEAP1; (C) Amphioxus (*Branchiostoma japonicum*) KEAP1.

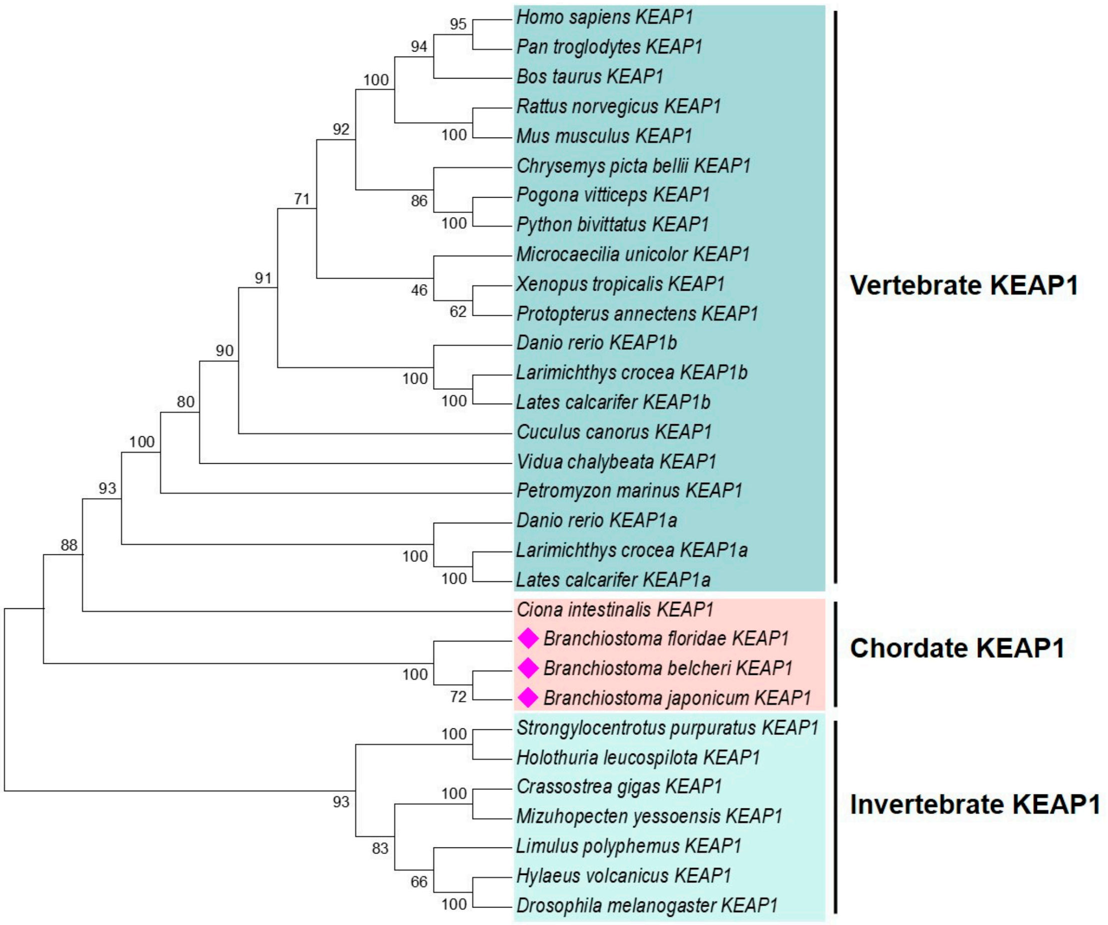


Figure S6. Phylogenetic tree of KEAP1 homologues. The phylogenetic tree was constructed by MEGA 7.0 using the amino acid-based Neighbor-Joining (NJ) algorithm. The reliability of each node was estimated by bootstrapping with 1000 replications. Amphioxus KEAP1 are shown in red background. Accession numbers for sequences used are listed in Table S3 of supplementary data.

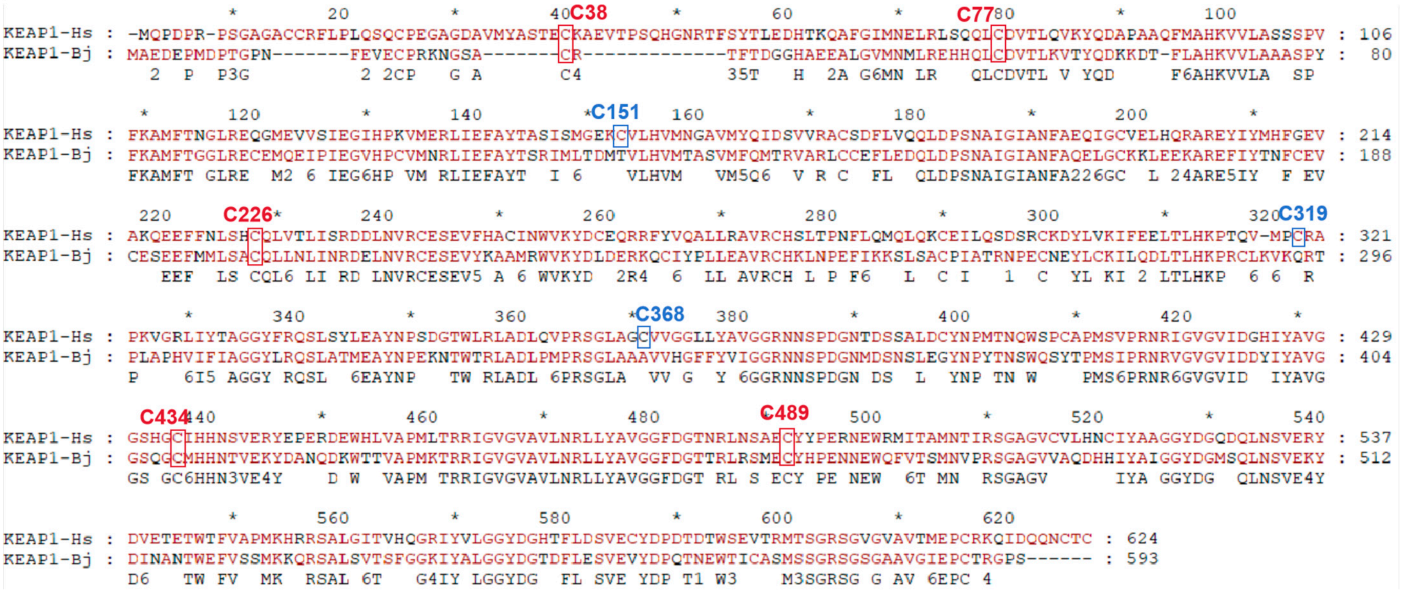


Figure S7. The eight most preferential cystine residues modified by sulforaphane in KEAP1. Amino acid alignments of *Homo sapiens* (Hs) and *Branchiostoma japonicum* (Bj) were performed using the MUSCLE program. Cysteine residues highlighted in red boxes indicate those conserved in both species, while those in blue boxes are absent in amphioxus.
